# Functional Gene Analysis Reveals Cell Cycle Changes and Inflammation in Endothelial Cells Irradiated with a Single X-ray Dose

**DOI:** 10.3389/fphar.2017.00213

**Published:** 2017-04-25

**Authors:** Bjorn Baselet, Niels Belmans, Emma Coninx, Donna Lowe, Ann Janssen, Arlette Michaux, Kevin Tabury, Kenneth Raj, Roel Quintens, Mohammed A. Benotmane, Sarah Baatout, Pierre Sonveaux, An Aerts

**Affiliations:** ^1^Radiobiology Unit, Belgian Nuclear Research Centre (SCK•CEN), Institute for Environment, Health and SafetyMol, Belgium; ^2^Institut de Recherche Expérimentale et Clinique (IREC), Pole of Pharmacology & Therapeutics, Université catholique de LouvainBrussels, Belgium; ^3^Faculty of Medicine and Life Sciences, Biomedical Research Institute, Hasselt UniversityHasselt, Belgium; ^4^Centre for Radiation, Chemical and Environmental Hazards, Public Health EnglandDidcot, UK; ^5^Biomedical Engineering Program and Department of Mechanical Engineering, University of South Carolina, Columbia, SC, USA; ^6^Department of Molecular Biotechnology, Ghent UniversityGhent, Belgium

**Keywords:** X-ray, endothelium, atherosclerosis, cardiovascular disease, cell cycle

## Abstract

**Background and Purpose:** Epidemiological data suggests an excess risk of cardiovascular disease (CVD) at low doses (0.05 and 0.1 Gy) of ionizing radiation (IR). Furthermore, the underlying biological and molecular mechanisms of radiation-induced CVD are still unclear. Because damage to the endothelium could be critical in IR-related CVD, this study aimed to identify the effects of radiation on immortalized endothelial cells in the context of atherosclerosis.

**Material and Methods:** Microarrays and RT-qPCR were used to compare the response of endothelial cells irradiated with a single X-ray dose (0.05, 0.1, 0.5, 2 Gy) measured after various post-irradiation (repair) times (1 day, 7 days, 14 days). To consolidate and mechanistically support the endothelial cell response to X-ray exposure identified via microarray analysis, DNA repair signaling (γH2AX/TP53BP1-foci quantification), cell cycle progression (BrdU/7AAD flow cytometric analysis), cellular senescence (β-galactosidase assay with CPRG and IGFBP7 quantification) and pro-inflammatory status (IL6 and CCL2) was assessed.

**Results:** Microarray results indicated persistent changes in cell cycle progression and inflammation. Cells underwent G1 arrest in a dose-dependent manner after high doses (0.5 and 2 Gy), which was compensated by increased proliferation after 1 week and almost normalized after 2 weeks. However, at this point irradiated cells showed an increased β-Gal activity and IGFBP7 secretion, indicative of premature senescence. The production of pro-inflammatory cytokines IL6 and CCL2 was increased at early time points.

**Conclusions:** IR induces pro-atherosclerotic processes in endothelial cells in a dose-dependent manner. These findings give an incentive for further research on the shape of the dose-response curve, as we show that even low doses of IR can induce premature endothelial senescence at later time points. Furthermore, our findings on the time- and dose-dependent response regarding differentially expressed genes, cell cycle progression, inflammation and senescence bring novel insights into the underlying molecular mechanisms of the endothelial response to X-ray radiation. This may in turn lead to the development of risk-reducing strategies to prevent IR-induced CVD, such as the use of cell cycle modulators and anti-inflammatory drugs as radioprotectors and/or radiation mitigators.

## Introduction

At higher doses of ionizing radiation (>0.5 Gy), epidemiological data show a significant excess risk of late occurring cardiovascular disease (CVD; Hildebrandt, 2010; Shimizu et al., 2010; Darby et al., 2013). The term CVD encompasses coronary heart disease and peripheral arterial disease, and is most often related to atherosclerosis (Lusis, 2000). In the clinics, these doses are used in 50 to 60% of all cancer patients during radiotherapy (Gottfried et al., 1996). During these treatments, normal surrounding tissues also receive a part of the dose. For example, the mean dose of radiation to the heart during breast cancer radiotherapy ranges from 0.03 to 27.72 Gy, with an overall average of 4.9 Gy (Darby et al., 2013). Still, knowledge of the underlying biological and molecular mechanisms of radiation-induced CVD is limited (Little et al., 2008; Hildebrandt, 2010) and should be complemented with experimental findings.

In addition to higher doses, low doses of ionizing radiation (≤0.1 Gy Fazel et al., 2009) are also increasingly used in the clinics, mainly for diagnostic medical purposes (UNSCEAR, 2008). Consequently, the possibility of an excess risk of CVD following exposure to ionizing radiation is of great societal concern. However, the current radiation protection system is based on the assumption that there is a threshold at 0.5 Gy where epidemiological evidence for non-cancer effects is suggestive (Hildebrandt, 2010). Indeed, over recent years, a growing body of epidemiological data suggested an excess risk of late occurring CVD at much lower doses, without a clear-cut threshold (Hildebrandt, 2010; Shimizu et al., 2010; Darby et al., 2013). However, due to a lack of statistical power, these data are suggestive rather than persuasive.

The mechanisms by which radiation causes CVD are at present unknown, but radiation acts, at least in part, by causing or promoting atherosclerosis (Borghini et al., 2013). Atherosclerosis is a multifactorial disease characterized by a chronic inflammatory process of the arterial wall. It is believed to be initiated by irritative stimuli (e.g., hypertension, dyslipidemia, and pro-inflammatory mediators) leading to endothelial dysfunction (Libby et al., 2011), a pathological state characterized by the loss of endothelial functions that are normally in place to maintain vascular integrity (Hirase and Node, 2012). This dysfunctional state induces a pro-inflammatory reaction in the endothelium, triggering the expression of adhesion molecules such as selectin P and intracellular adhesion molecule 1 (ICAM1; Collins et al., 2000) and the secretion of cytokines such as C-C Motif Chemokine Ligand 2 (CCL2; Boring et al., 1998) and interleukin-6 (IL6) (Schieffer et al., 2004). These molecules mediate the attachment of circulating monocytes and lymphocytes, driving the intimal immune cell infiltration. Eventually, a chronic inflammatory reaction ensues, resulting in the formation of an atheroma filled with foam cells and a necrotic core (Weber and Noels, 2011). By using bioinformatics and metanalytical approaches, knowledge on the molecular networks that are common in ionizing radiation, immune and inflammatory responses is emerging (Georgakilas et al., 2015). These studies will further help to understand the underlying molecular mechanisms of radiation-induced inflammatory reactions and will help to explain the pathogenesis of radiation-induced CVD.

In the radiobiological context, endothelial cells have been proposed to be a critical target in radiation-induced CVD (Little et al., 2008; Hildebrandt, 2010). Indeed, exposure of the vascular endothelium to IR can result in endothelial cell dysfunction (Bhattacharya and Asaithamby, 2016), a well-established cardiovascular risk factor that promotes the development and progression of atherosclerosis (Vita and Keaney, 2002; Widmer and Lerman, 2014). Although the mechanisms of radiation-induced CVD are far from being understood, inflammatory processes seem to be involved. Following irradiation, upregulation of several pro-inflammatory molecules by endothelial cells has been observed. For example, the expression of ICAM1 and selectin P increased after irradiation in both *in vitro* and *in vivo* experiments (Gallo et al., 1997; Hallahan and Virudachalam, 1997a,b; Van Der Meeren et al., 1999; Haubner et al., 2013). Furthermore, endothelial cells upregulate the secretion of several pro-inflammatory cytokines, such as IL6 and CCL2, after irradiation (Van Der Meeren et al., 1999; Haubner et al., 2013).

In this study, we tried to find molecular evidence for the presence of an excess risk of CVD following exposure of endothelial cells to low single X-ray doses (0.05 and 0.1 Gy), a caveat in current radiobiological knowledge. Furthermore, we aimed to identify underlying biological and molecular mechanisms of radiation-induced CVD after exposure of endothelial cells to a single X-ray dose (0.05, 0.1, 0.5, 2 Gy). Compared to the existing knowledge, our study looks at longer time spans after radiation exposure combined with the use of human coronary artery endothelial cells. These endothelial cells are linked to coronary artery disease, observed after radiation exposure during radiotherapy in females with breast cancer (Darby et al., 2013). Endothelial cells were irradiated with a single X-ray dose (0.05, 0.1, 0.5, 2 Gy) and transcriptomic changes were measured after various post-irradiation (repair) times (1 day, 7 days, 14 days). We report that a single X-ray dose induces dose- and time-dependent transcriptional changes associated with atherosclerosis-related processes in immortalized human coronary artery endothelial cells.

## Materials and methods

### Cells and irradiation

Human telomerase-immortalized coronary artery endothelial (TICAE) cells (ECACC) were grown in Human MesoEndo Endothelial Cell Medium (Cell Applications) and cultured at 37°C with 5% CO_2_ in a humidified incubator as described elsewhere (Lowe and Raj, 2014). Cells were irradiated at >95% confluence with a dose rate of 0.50 Gy/min, using an AGO HS320/250 X-ray cabinet (only for microarray samples; 250 kV, 13 mA, 1.5 mm Al, and 1.2 mm Cu) or an Xstrahl RX generator (for validation samples; 250 kV, 12 mA, 3.8 mm Al, and 1.4 mm Cu). Cells were not passaged during experiments, but medium was changed thrice per week.

### Microarrays

Total RNA of TICAE cells was extracted according to manufacturer's instructions using the AllPrep DNA/RNA/protein mini kit (Qiagen). RNA was quantified using a NanoDrop Spectrophotometer and its quality assessed with an Agilent 2100 Bioanalyzer. Samples with a RNA integrity number >8 were used for hybridization onto Affymetrix Human Gene 2.0 ST arrays, following manufacturer's instructions. Raw data were uploaded to the Partek Genomics Suite (version 6.6) and normalized using a customized Robust Multi-chip Analysis algorithm (background correction for entire probe sequence, quantile normalization, log2 transformation of intensity signals). Data are available in the ArrayExpress database (http://www.ebi.ac.uk/arrayexpress; accession number E-MTAB-5054).

### Functional enrichment analysis

Functional gene enrichment was performed and visualized using GOrilla (Eden et al., 2007, 2009). Settings were: organism: *Homo sapiens*; running mode: two unranked lists of genes (target list: differentially expressed genes, background list: genes expressed above background in at least 33% of all samples); *P*-value threshold: 0.001. To exclude redundant gene ontology terms, results were reduced using REViGO (Rudjer Boskovic Institute, Croatia) with an authorized similarity of 0.4 (Supek et al., 2011). Gene Ontology version used was go_201507-termdb.obo-xml.gz (http://archive.geneontology.org/full/2015-07-01/).

### Transcription factor binding site enrichment analysis

Chromatin immunoprecipitation (ChIP) enrichment analysis was performed with Enrichr (Icahn School of Medicine at Mount Sinai, USA) to identify transcription factors that were enriched for target genes within the list of differentially expressed genes (Chen et al., 2013; Kuleshov et al., 2016). Databases from all species, cell types and ChIP methods were interrogated.

### Reverse transcription quantitative PCR (RT-qPCR)

RT-qPCR analysis was performed as previously described (Verreet et al., 2015) on a 7,500 Fast Real-Time PCR system (Applied Biosystems). Gene expression was normalized to reference genes *RAP2C* and *INPP1*. Gene expression ratios were calculated using the Pfaffl method (Pfaffl, 2001). Data were normalized to the values of the respective control samples at the same time point (either 1 day, 7 days, 14 days) and presented as the average expression ratio.

### DNA double strand break repair kinetics

To identify DNA double strand breaks (DSBs) and early DNA damage repair response, cells were stained for phosphorylated histone H2AX (γH2AX) and tumor suppressor p53-binding protein 1 (TP53BP1). After irradiation, cells were fixed (2% PFA), permeabilized (0.25% Triton X-100 in PBS), blocked (1% normal goat serum [Themo Fisher] in Tris-NaCl [Perkin Elmer]) and probed with primary anti-γH2AX (1/300; Merck-Millipore #05-636) and anti-TP53BP1 (1/1,000; Novus Biologicals #NB100-304) antibodies (1 h, 37°C). After washing, cells were incubated with 1 μg/ml DAPI and secondary Alexa Fluor 488 and 568 (Life technologies) antibodies (1 h, 37°C). Cells on slides were visualized with an Eclipse Ti microscope (NIKON) equipped with a 40 × Plan Fluor objective (NA 0.6) and an Andor Ixon EMCCD camera. Twelve fields (z-stack of 9 planes axially separated by 1 μm) were captured per replicate with a lateral spacing of 500 μm. Images were analyzed with FIJI software (Schindelin et al., 2012) using the Cellblocks toolbox (De Vos et al., 2010). In brief, software allowed to analyze each nucleus based on the DAPI signal using Gaussian filtering and region of interest identification. Within each nucleus, pixel size and intensity emitted from the Alexa 488 (γH2AX) and Alexa 568 (TP53BP1) fluorochromes along with their overlap were analyzed, after which the foci number per nucleus was determined in a fully automatic manner using a predefined threshold algorithm combined with multi-scale Laplacian filtering. Settings used were triangle threshold algorithm with a Laplacian scale of 2 and a minimum foci size of 3 pixels. Six biological replicates were screened per condition, and a minimum of 200 nuclei were analyzed per replicate.

### Cell cycle

TICAE cells were treated with 10 μM of BrdU for 1 h, followed by ethanol fixation for a minimum of 24 h. Cells were permeabilized and stained with rat anti-BrdU antibody (AbD Serotec, #OBT0030CX) and 10 μg/ml 7-amino-actinomycin D (Sigma-Aldrich). Samples were run on a BD Accuri C6 flow cytometer, with a maximum flow speed of 300 events/s.

### Senescence

To determine senescence-associated β-galactosidase activity, cells were lysed in M-PER reagent (Thermofisher Scientific). Lysates were incubated at 37°C for 18 h in reaction buffer (1 mM MgCl_2_, 2 mM chlorophenolred-β-D-galactopyranoside in 50 mM phosphate buffer, pH 6.0). Reaction was stopped by adding 1 M of Na_2_CO_3_, and absorbance was measured at 570 nm.

### Multiplex bead array

C-C motif chemokine 2 (CCL2), interleukin 6 (IL6) and insulin-like growth factor binding protein 7 (IGFBP7) levels in cell culture supernatants were analyzed using a multiplex magnetic bead array (R&D systems). Assays were performed according to manufacturer's instructions. Samples were run on a Luminex 200 and analyzed with xPONENT 3.1 (Luminex Corporation).

### Statistics

Data show means ± SEM. Microarray data were filtered to exclude genes expressed below the background signal in at least 67% of all samples and analyzed using two-way ANOVA. Differentially expressed genes were identified as those with a fold change >|1.5| and *P* < 0.05 after correction for multiple testing according to Benjamini and Hochberg (Benjamini and Hochberg, 1995). Other data were analyzed using two-way ANOVA with Bonferroni *post-hoc* test. *P* < 0.05 was considered statistically significant.

## Results

### Irradiation with 0.5 and 2 Gy alters gene expression in endothelial cells

Amongst other cardiovascular effects, radiation exposure has been shown to accelerate age-related atherosclerosis leading to coronary artery disease (Darby et al., 2013). Since endothelial cell dysfunction is a well-established risk factor of atherosclerosis (Vita and Keaney, 2002; Widmer and Lerman, 2014), we tested whether irradiation of endothelial cells with a single X-ray dose (0.05, 0.1, 0.5, 2 Gy) measured after various post-irradiation (repair) times (1 day, 7 days, 14 days) could activate pro-atherosclerotic processes *in vitro*. We observed dose- and time-dependent changes in gene expression using a genome-wide gene expression analysis. Differentially expressed genes are listed in Supplementary Tables 1–3, and their number is shown in Table 1. Single X-ray doses of 0.05 and 0.1 Gy did not affect gene expression at any of the investigated time points. In contrast, a single X-ray dose of either 0.5 or 2 Gy induced marked and comparable (Supplementary Figure 1) differences in gene expression detected on day 1 post-irradiation. While the response was essentially transient at 0.5 Gy, a large number of genes were still differentially expressed in TICAE cells 7 and 14 days after irradiation with a single X-ray dose of 2 Gy.

**Table 1:** Irradiation with a single dose of X-rays induces differential gene expression in TICAE cells^*^.

	**1 day**	**7 days**	**14 days**
0.05 Gy	0	0	1 (−1)
0.1 Gy	0	0	1 (−1)
0.5 Gy	162 (+22/−140)	2 (+1/−1)	4 (−4)
2 Gy	522 (+107/−415)	129 (+79/−50)	59 (+41/−18)

* *TICAE cells were analyzed at the indicated time points after irradiation with a single dose of X-rays. Changes are shown compared to sham irradiation, as described in Materials and Methods (n = 3). Numbers between brackets indicate up (+) and/or down (−) regulated genes per condition*.

### Irradiation of endothelial cells represses the expression of genes involved in cell cycling and induces inflammation

**Figure 1:**
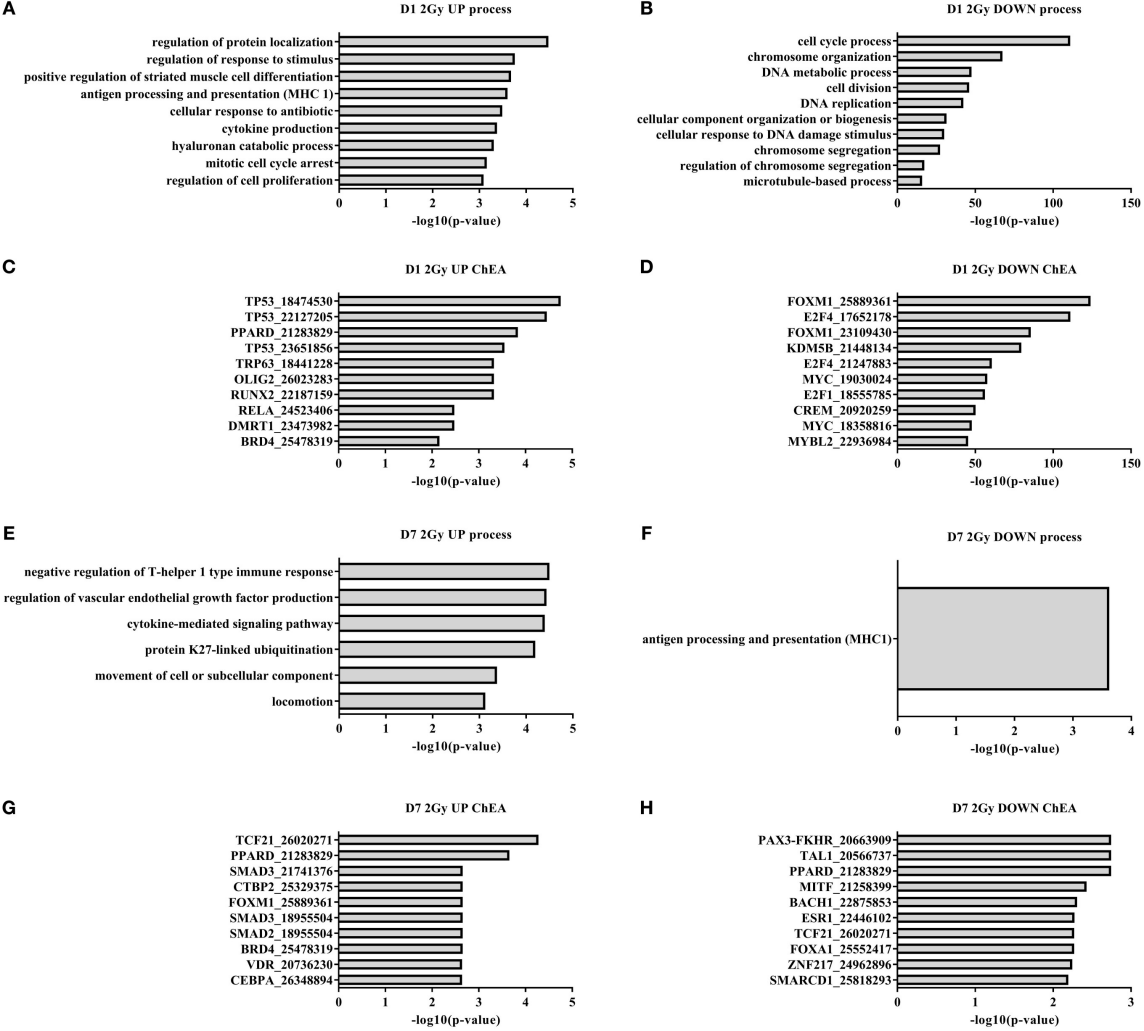
**Irradiated TICAE cells differentially express genes controlling cell cycling and inflammation**. Gene ontology and ChIP enrichment analysis (ChEA) was performed on differentially expressed genes in irradiated vs. sham-irradiated TICAE cells. (*n* = 3). **(A)** Top 10 GO enrichment terms among upregulated differentially expressed genes 1 day after irradiation with a single X-ray dose of 2 Gy. **(B)** As in **(A)** but showing downregulated differentially expressed genes. **(C)** Top 10 enriched predicted upregulated transcriptional regulators in TICAE cells 1 day after irradiation with a single X-ray dose of 2 Gy. **(D)** As in **(C)** but showing downregulated transcriptional regulators. **(E)** As in **(A)** but at 7 days post-irradiation. **(F)** As in **(B)** but at 7 days post-irradiation. **(G)** As in **(C)** but at 7 days post-irradiation. **(H)** As in **(D)** but at 7 days post-irradiation. PubMed ID numbers are included in the ChIP enrichment analysis graphs. Enrichment was scored using FDR-corrected *P*-value. Full gene ontology and ChIP enrichment analysis results are listed in the Supplementary data files.

Functional enrichment analysis revealed that, 1 day after exposure to a single X-ray dose of 2 Gy, the upregulated differentially expressed genes were involved in cell cycle arrest and cytokine production and the downregulated ones in cell cycle-related processes such as mitotic cell cycle, chromosome organization and microtubule dynamics (Figures 1A,B). ChiP enrichment analysis was then used to identify transcription factors that control sets of differentially expressed genes. The identified transcription factors were shown to regulate cell cycle responses to DNA damage [such as p53 (Lane, 1992), Myc (Wasylishen and Penn, 2010), FOXM1 (Zona et al., 2014) and members of the E2F family (Bertoli et al., 2013)] and cytokine production (RELA and NF-κB; Magné et al., 2006) (Figures 1C,D).

At day 7 and 14 after exposure to a single X-ray dose of 2 Gy, 129 and 59 genes were differentially expressed, respectively (Table 1). On day 7, gene ontology analysis highlighted inflammation-related processes, including induction of the negative regulation of T-helper 1 type immune response, cytokine-mediated signaling and suppression of MHC1 antigen processing and presentation (Figures 1E,F). ChiP enrichment analysis identified several inflammation-associated transcription factors (Figures 1F,G), among which PPARδ, known to regulate multiple pro-inflammatory pathways (Barish et al., 2008), endothelial cell proliferation and angiogenesis (Piqueras et al., 2007) and SMAD2/3, involved in TGFβ signaling (Derynck and Zhang, 2003). Furthermore, transcription factors vitamin D receptor, which promotes autophagy and cell survival pathways (Uberti et al., 2014), and BACH1, involved in oxidative stress response and cell-cycle progression (Wang et al., 2016a), were identified. Although not listed in the top 10, members of the E2F and p53 families of transcription factors were also identified (Supplementary data files), indicating a persistent cell cycle response. On day 14 following the exposure to a single X-ray dose of 2 Gy, no enrichment of transcription factors or gene ontology terms was identified.

### Ionizing radiation causes a dose-dependent repression of the expression of genes controlling mitotic endothelial cell proliferation

**Figure 2:**
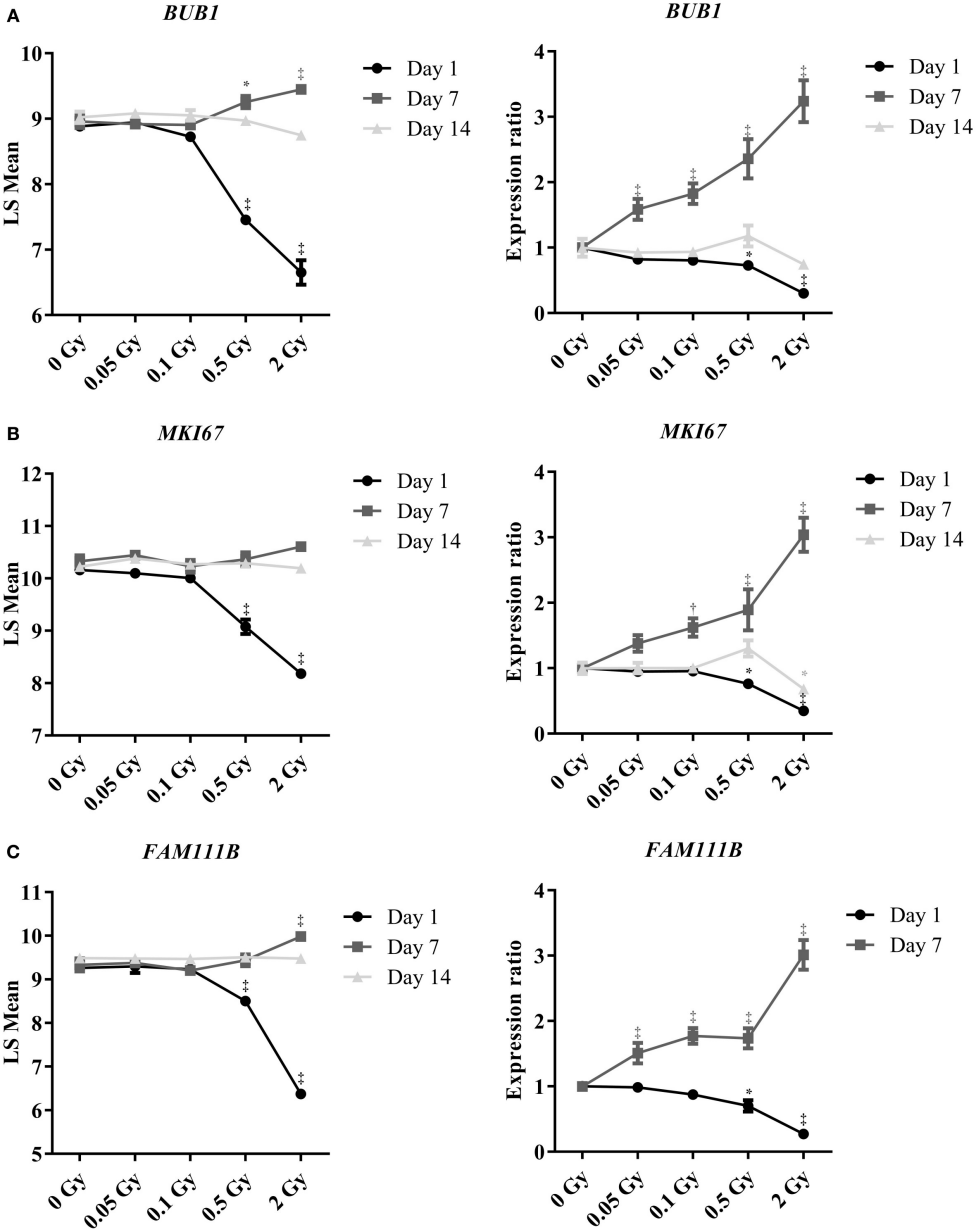
**Irradiation induces dose- and time-dependent repression of the expression of genes controlling mitotic endothelial cell proliferation**. Graphs show changes in gene expression of **(A)** *BUB1*, **(B)** *MKI67* and **(C)** *FAM111B*. Left graphs show data obtained using microarrays (*n* = 3) and right graphs using RT-qPCR (*n* = 6). Of note, *FAM111B* expression was below detection threshold by RT-qPCR on day 14 post-irradiation. ^*^P < 0.05, ^†^*P* < 0.005, ^‡^*P* < 0.001 compared to sham, using 2-way ANOVA with Bonferroni *post-hoc* test.

To confirm the effect of a single X-ray dose on the proliferation of endothelial cells, as observed in the microarray data, we performed RT-qPCR analysis on independent samples. *BUB1, FAM111B* and *MKI67* genes were selected for their involvement in mitotic cell cycle progression: *BUB1* is a mitotic checkpoint kinase (Bolanos-Garcia and Blundell, 2011), *MKI67* encodes proliferation marker Ki67 (Gerdes et al., 1983) and *FAM111B* has an unknown function but could be related to DNA replication (Aviner et al., 2015). Microarray and RT-qPCR data matched for *BUB1* (Figure 2A), *MKI67* (Figure 2B) and *FAM111B* (Figure 2C), with a significant decrease in gene expression at a single X-ray dose of 0.5 or 2 Gy on day 1 post-irradiation, followed by a slight increase on day 7 and a return to basal expression on day 14. However, contrary to microarray results, RT-qPCR indicated a dose-dependent increase in the expression of all three genes at all irradiation doses on day 7 post-irradiation. This could be due to differences in the normalization technique used in the assays (Morey et al., 2006).

### Radiation exposure activates DNA damage signaling, induces an acute G1 arrest and leads to premature senescence

**Figure 3:**
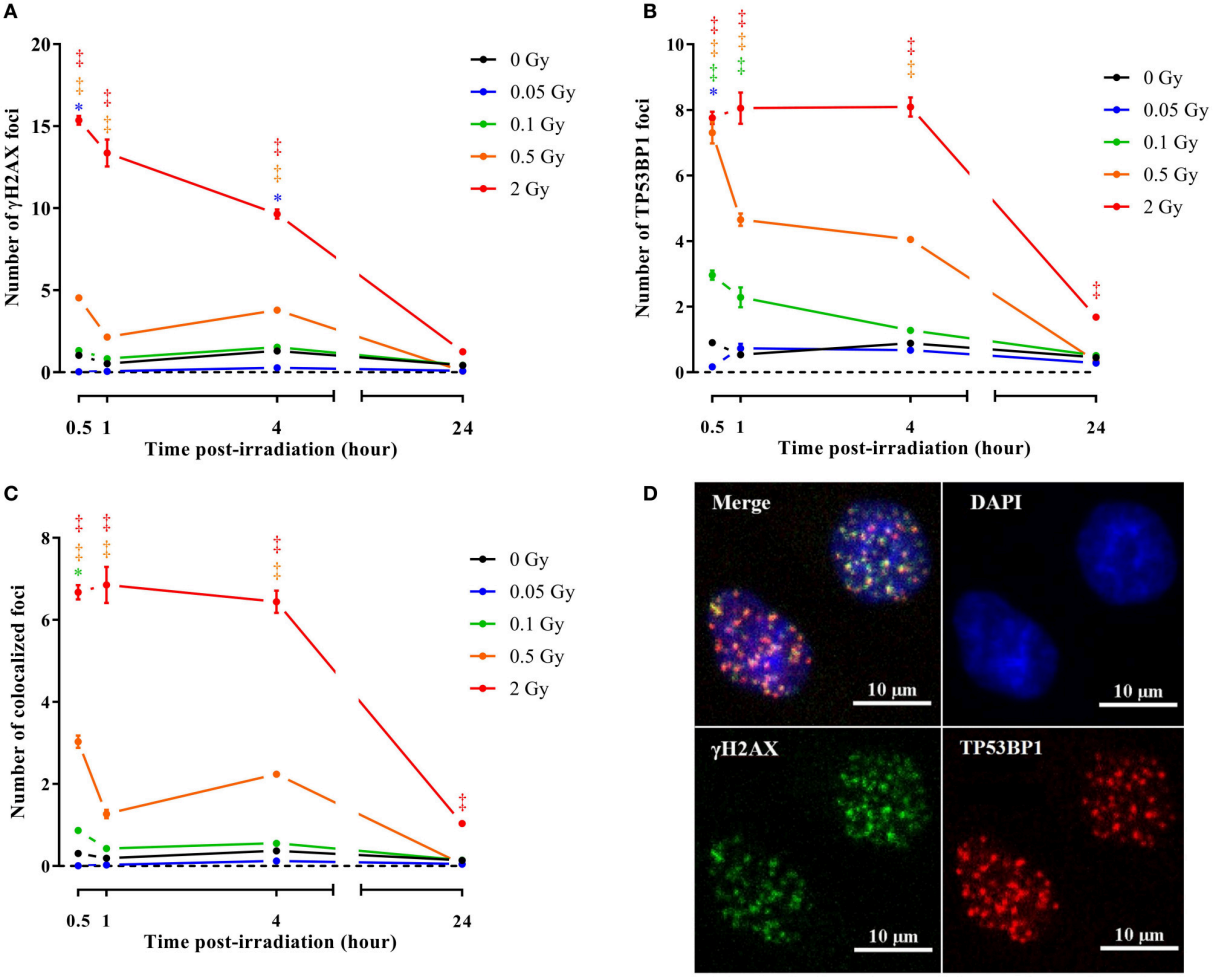
**Irradiation induces endothelial DNA damage signaling**. Graphs represent the amount of γH2AX **(A)**, TP53BP1 **(B)** and colocalized γH2AX/TP53BP1 **(C)** foci 30 min, 1, 4, and 24 h after irradiation with a single X-ray dose of 0.05, 0.1, 0.5 and 2 Gy (*n* = 6). Data show means ± SEM. ^*^P < 0.05, ^†^*P* < 0.005, ^‡^*P* < 0.001 compared to sham on the same day, using 2-way ANOVA with Bonferroni *post-hoc* test. **(D)** Representative images showing γH2AX (green), TP53BP1 (red) and γH2AX+TP53BP1 (yellow) foci in DAPI stained nuclei (blue) of TICAE cells 30 min after irradiation with a single X-ray dose of 2 Gy.

**Figure 4:**
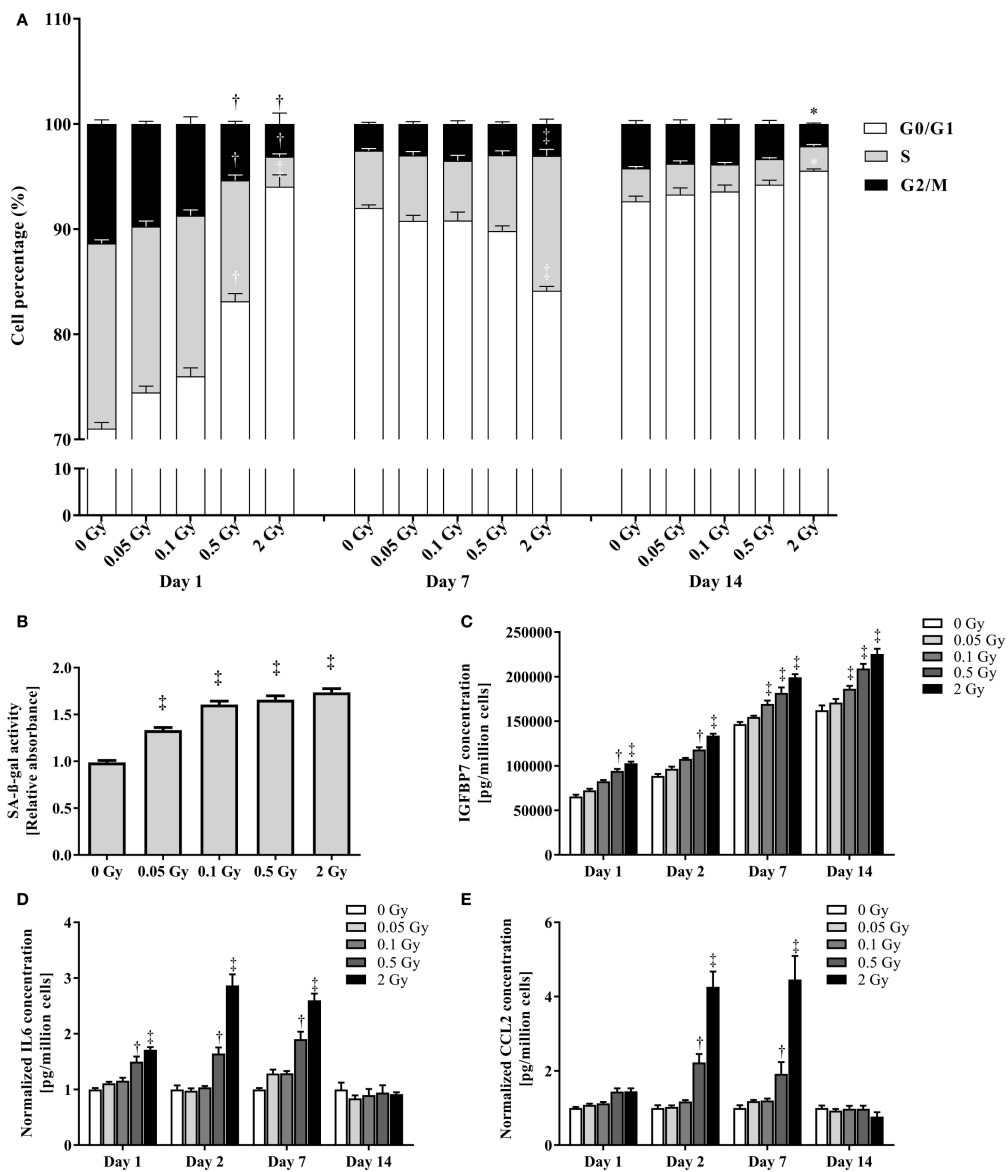
**Irradiation reversibly inhibits cell cycle progression, induces premature endothelial senescence and promotes endothelial cell inflammation. (A)** The histogram represents the % of TICAE cells in the different phases of the cell cycle at the indicated times after irradiation with the indicated doses (*n* = 6). **(B)** The histogram represents the activity of senescence-associated β-galactosidase (SA-β-gal) 14 days after irradiation at the indicated doses (*n* = 16). Data are normalized to cell numbers and control values. **(C,D,E)** Histograms represent the amount of IGFBP7 **(C)**, IL6 **(D)** and CCL2 **(E)** secreted by TICAE cells at the indicated times after irradiation at the indicated doses (*n* = 8). Data are normalized to cell numbers alone **(C)** or to cell numbers and control values **(D,E)**. Data show means ± SEM. ^*^P < 0.05, ^†^*P* < 0.005, ^‡^*P* < 0.001 compared to sham on the same day, using 2-way ANOVA with Bonferroni *post-hoc* test.

To consolidate and mechanistically support the endothelial cell response to a single X-ray dose exposure identified during microarray analysis, we performed immunocytochemical stainings for γH2AX and TP53BP1, two markers of DNA DSB repair (Wang et al., 2014) that interact during DNA damage response and are linearly related to DSB number and radiation dose (Wang et al., 2014; Kleiner et al., 2015). Irradiation with a single X-ray dose rapidly and dose-dependently increased the number of γH2AX, TP53BP1 and colocalized γH2AX+TP53BP1 foci (Figures 3A–D, Supplementary Figures 2–5). Foci numbers were maximal 30 min to 1 h after irradiation, followed by an almost complete decline at 24 h, except for 2 Gy where residual foci were probably indicative of lethal DNA damage (Banáth et al., 2010). To study the effect of a single X-ray dose on cellular proliferation, we performed a cell cycle analysis to study the cell cycle progression. On day 1 after irradiation, we detected a dose-dependent increase in the percentage of cells in G0/G1 and a decrease in the percentage of cells in S and G2/M phases, indicating that the cells arrested at the G1 checkpoint (Figure 4A). On day 7 post-irradiation, cells reached a state of contact inhibition and were mostly in G0/G1 in the sham condition. Irradiated cells displayed a cell cycle profile similar to that of control cells, except for the 2 Gy dose, where significantly more cells were in S phase and less in G0/G1, indicating higher cellular proliferation. Increased proliferation was presumably due to the absence of contact inhibition resulting from cell death, as well as a longer and stronger G1 arrest induced at day 1. On day 14 post-irradiation, we detected no difference for sham-irradiated cells vs. cells irradiated with a single dose of 0.05, 0.1 and 0.5 Gy. Compared to sham, endothelial cells irradiated with a single X-ray dose of 2 Gy still had a disturbed cell cycle progression with more cells in G0/G1 and less in G2/M, which may be indicative of premature senescence. In accordance, all radiation doses increased senescence-associated β-galactosidase activity (Dimri et al., 1995; Figure 4B) and insulin-like growth factor-binding protein 7 (IGFBP7) secretion (Wajapeyee et al., 2008; Figure 4C) in TICAE cells on day 14 post-irradiation.

### Irradiation induces IL6 and CCL2 expression in endothelial cells

To confirm the induction of inflammation in irradiated TICAE cells, we focused on two pro-inflammatory cytokines involved in atherosclerosis: IL6 (Schuett et al., 2009) and CCL2 (Harrington, 2000). On days 1, 2 and 7 post-irradiation, IL6 secretion was significantly increased in endothelial cells irradiated with a single X-ray dose of 0.5 and 2 Gy (Figure 4D), while CCL2 secretion increased on days 2 and 7 post-irradiation following a single X-ray dose of 0.5 and 2 Gy (Figure 4E). Both pro-inflammatory cytokines returned to control levels after 14 days, thus indicating the presence of transient radiation-induced inflammation.

Altogether, gene expression, cell cycle and cytokine analysis unraveled an altered proliferation and an increased inflammatory state in endothelial cells at 1, 2, and 7 days after exposure to a single X-ray dose. At 14 days post-irradiation, cellular proliferation and inflammatory state reverted back to levels similar to those observed in non-irradiated (control) samples. However, at this time point the endothelial cells irradiated with a single X-ray dose, ranging from 0.05 to 2 Gy, demonstrated an increased senescence-associated-β-galactosidase activity and IGFBP7 secretion, indicative of premature senescence at all doses under investigation.

## Discussion

**Figure 5:**
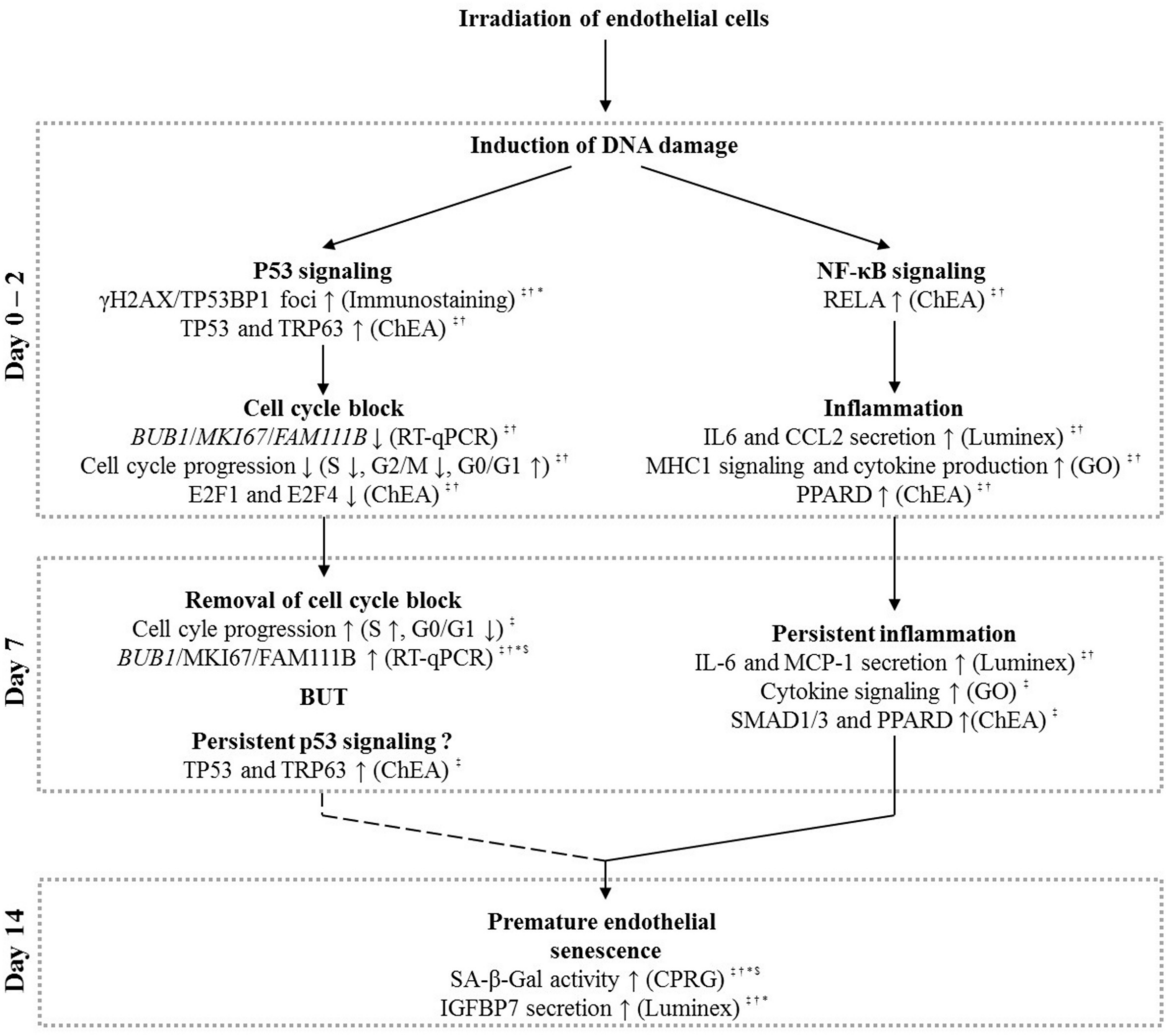
**Schematic overview based on our experimental findings and literature that explains the possible role of p53 and NF-κB signaling in radiation-induced premature senescence**. Irradiation of endothelial cells with a single X-ray dose leads to the formation of DNA damage, resulting in both p53 and NF-κB signaling, well-known signaling responses in irradiated cells (Yu, 2012). At day 0–2 post-irradiation, induced p53 signaling can result in a G1/S cell cycle block (Agarwal et al., 1995), which is removed at day 7 post-irradiation. Although not conclusive (question mark symbol), we also suggest the presence of a persistent p53 signaling at this time-point. NF-κB signaling on day 0–2 post-irradiation, on another hand, could induce endothelial inflammation (Kempe et al., 2005) that persists until day 7 post-irradiation. Both the persistent p53 signaling (Rufini et al., 2013) and inflammatory state (Freund et al., 2010; Kojima et al., 2013) may lead to premature senescence observed at day 14 after irradiation with a single X-ray dose. Symbols indicate the irradiation dose at which the described effects were observed: ^‡^ = 2 Gy, ^†^ = 0.5 Gy, ^*^ = 0.1 Gy, and ^$^ = 0.05 Gy.

Our study aimed at investigating whether endothelial cell irradiation induced pro-atherosclerotic processes as suggested by epidemiological data (Shimizu et al., 2010; Darby et al., 2013). Furthermore, we aimed to determine whether low doses (0.05 and 0.1 Gy; doses received during serial diagnostics) of ionizing radiation could induce the same effects as those observed at higher doses (0.5 and 2 Gy; doses received on healthy tissues during radiotherapy) of ionizing radiation. We report dose- and time-dependent repression of endothelial cell cycling with an increased senescence-associated β-galactosidase activity and inflammatory cytokine secretion (Figure 5). These changes are indicative of a pro-atherosclerotic phenotype in endothelial cells.

A predominant effect of IR exposure is cell cycle arrest. Accordingly, in endothelial cells we showed that irradiation with a single X-ray dose ≥0.5 Gy represses the expression of genes that support cell cycle progression. We evidenced a G1 arrest 1 day after single dose irradiation with 0.5 and 2 Gy of X-rays. G1 arrest generally results from p53 activation in response to DNA damage (Bernhard et al., 1995), which was evidenced with enrichment analysis and the formation of γH2AX and TP53BP1 foci. To determine the complexity of the DNA damage after exposure to ionizing radiation, future studies should look at the colocalization between DSB and non-DSB damage (Nikitaki et al., 2016). G1 arrest was followed by a progressive restoration of cell cycling for all doses, except for 2 Gy where cell cycling restoration was followed by an additional G1 arrest, which coincided with increased senescence-associated β-galactosidase activity and IGFBP7 secretion. Thus, exposure to a single X-ray dose induced markers linked to senescence, even at a dose as low as 0.05 Gy. Persistent p53 family signaling due to persistent DNA damage, as seen in our enrichment analysis at day 7, could be responsible for premature senescence (Rufini et al., 2013). Our findings at low doses of X-rays are consistent with previous studies having evidenced premature senescence at higher doses in the same cell line (Lowe and Raj, 2014) and upon chronic exposure to low dose rates in different endothelial cells (Yentrapalli et al., 2013a,b; Rombouts et al., 2014). Senescent endothelial cells are shown to be present in human atherosclerotic plaques (Minamino et al., 2002) and are emerging as a contributor to the pathogenesis of atherosclerosis (Wang et al., 2016b). Therefore, premature senescence could play a role in radiation-induced CVD (Wang et al., 2016b), and research with cell cycle modulators as radioprotectors is warranted. However, there is no universal marker or hallmark of senescence identified so far that is entirely specific to the senescent state of a cell. Furthermore, not all senescent cells express all the possible senescence markers identified so far. As a consequence, future studies are needed to corroborate our *in vitro* findings in other *in vitro* as well as *in vivo* models. These studies should use highly sensitive techniques adapted to determine the presence of endothelial cell senescence even at low doses of ionizing radiation. One of such innovative and highly sensitive dyes is Sudan-Black-B, a specific lipofuscin stain that can be used to detect both replicative and stress-induced senescence independently of sample preparation (Evangelou et al., 2017).

Inflammation plays a pivotal role in the development, progression and final outcome of atherosclerosis (Libby, 2002). In this study, we evidenced radiation-induced inflammation in endothelial cells after exposure to a single X-ray dose of 0.5 and 2 Gy. Although the cause of this pro-inflammatory reaction is not entirely clear, the release of damage-associated molecular patterns (DAMPs) by stressed and apoptotic cells could be involved. One of the DAMPs released by apoptotic cells, called HMGB1, has been described to induce IL6 and CCL2 secretion as well as the expression of ICAM1 and VCAM1 in endothelial cells (Sun et al., 2013). Binding of these DAMPs to Toll-like receptors on endothelial cells upregulates pro-inflammatory signaling molecules such as NF-κB (Fiuza et al., 2003). In accordance, transcriptional regulation by pro-inflammatory factors RELA and PPARδ, negative regulation of the T-helper 1 type immune response, suppression of MHC1 antigen processing and presentation, and cytokine-mediated signaling were detected with enrichment analyses. Inflamed endothelial cells initiate the formation of atherosclerotic plaques by attracting leukocytes and enabling their extravasation and migration (Libby, 2002). After activation, leukocytes form a dynamic and multilateral relationship with the vascular wall cells, ultimately driving and determining the course of the disease (Libby, 2002). Accordingly, we detected increased levels of pro-atherosclerotic and pro-inflammatory cytokines IL6 and CCL2 in endothelial cells after irradiation with a single X-ray dose of 0.5 and 2 Gy. IL6 contributes to plaque initiation and destabilization via a variety of mechanisms that include the release of other pro-inflammatory cytokines, prothrombotic mediators, acute phase reactants and lipoprotein oxidation (Schuett et al., 2009). CCL2 recruits monocytes into the subendothelial cell layer, a key step in the initiation and development of plaques (Harrington, 2000). Similar to our data, other endothelial cell types were found to produce significant amounts of pro-inflammatory molecules such as IL6, IL8, ICAM1 and VCAM1 following irradiation with a single X-ray dose of 2 Gy (Van Der Meeren et al., 1999; Haubner et al., 2013). However, these studies did not address long-term effects of radiation exposure. The significance of an inflammatory reaction in irradiated endothelial cells has yet to be determined as it could induce adverse effects on the vascular function (Hingorani et al., 2000; Paoletti et al., 2004). In this context, previous *in vivo* experiments indicated that irradiation predisposes ApoE^−/−^ obese mice to atherosclerotic plaques with an inflammatory phenotype prone to hemorrhage (Stewart et al., 2006). These prothrombotic changes may also accelerate atherosclerosis (Hoving et al., 2012). Interestingly, inflammation has also been identified as a cause of cellular senescence (Freund et al., 2010), and IL6 has been shown to induce senescence in fibroblasts through IGFBP-related processes (Kojima et al., 2013). We found similar responses in endothelial cells.

The current study used telomerase-immortalized human coronary artery endothelial cells. These endothelial cells are not tumorigenic (unpublished data), were shown to have a similar response to IR compared to their primary counterparts (Lowe and Raj, 2014) and display all major endothelial phenotypic markers, such as von Willebrand factor, PECAM1 and cadherin-5 (unpublished data). However, one must stress that our *in vitro* study did not integrate several biological aspects related to the complexity of the development of radiation-induced CVD in humans. For example, not only macrovascular (e.g., coronary arteries) but also microvascular injury could be at the basis of radiation-induced CVD (Darby et al., 2010). In the context of the ProCardio FP7 project, our findings will be integrated with other *in vitro, in vivo* and epidemiological data to increase our understanding of radiation-induced CVD.

In conclusion, we found that exposure of endothelial cells to a single X-ray dose induces a dose- and time-dependent cell cycle arrest, senescence and inflammation. These findings are indicative of the activation of pro-atherosclerotic processes and bring insights into the underlying molecular mechanisms of the endothelial response to X-ray irradiation. Although we cannot conclude that there is no threshold effect of irradiation-induced cardiovascular risk, our findings give an incentive for further research on the shape of the dose-response curve. Future research should also explore fractionated radiotherapy in its clinical mode and should further investigate the link between the possible release of DAMPs by irradiated endothelial cells and their response to radiation. Elucidation of the role of inflammation and premature senescence after radiation exposure, their timing and their involvement in the onset, progression and outcome of human atherosclerosis is now warranted in order to optimize the radiation protection system and to devise cardiovascular risk-reducing strategies if necessary. Optimization could be sought in the field of modern radiotherapy techniques, which reduce the dose to the normal tissues (MacDonald et al., 2013; Beck et al., 2014; Ngwa et al., 2014), and radiation protectants or mitigators that could reduce the deleterious effects of irradiation on the normal tissues (Raviraj et al., 2014).

## Author contributions

BB performed microarray analysis (functional enrichment analysis and transcription factor binding site enrichment analysis), RT-qPCR experiments, cell cycle analysis, senescence assays and multiplex bead array analysis. Furthermore, he performed (statistical) analysis of all the data provided in this manuscript. NB helped BB during RT-qPCR experiments, DNA double strand break repair assays, cell cycle analysis and senescence assays. EC stained and quantified DNA double strand break repair kinetics. DL and KR created and validated the human coronary artery endothelial cell line. Microarrays were performed by AJ and AM. KT performed multiplex bead arrays. RQ assisted during microarray analysis (functional enrichment analysis and transcription factor binding site enrichment analysis) and RT-qPCR primer design. BB, NB, EC, DL, KT, KR, RQ, MB, SB, PS, and AA helped with data interpretation, scientific guidance and preparation of the manuscript.

## Funding

This work was funded by EU FP7 DoReMi network of excellence (grant #249689), EU FP7 project ProCardio (grant #295823), the Belgian Federal Agency for Nuclear Control FANC-AFCN (grant #CO-90-13-3289-00) and the Belgian Fonds National de la Recherche Scientifique (F.R.S.-FNRS). BB, NB, and EC are supported by a doctoral SCK•CEN grant. PS is a F.R.S.-FNRS Senior Research Associate. Funding sources had no role in study design, data collection analysis and interpretation, the writing and submission of the manuscript for publication.

### Conflict of interest statement

The authors declare that the research was conducted in the absence of any commercial or financial relationships that could be construed as a potential conflict of interest.

## Supplementary material

Supplementary Data Sheet 1 **Irradiated TICAE cells differentially express genes controlling cell cycling and inflammation.** ChIP enrichment analysis (ChEA) was performed on differentially expressed genes in irradiated versus sham-irradiated TICAE cells (*n* = 3). Full list of the enriched predicted upregulated and downregulated transcriptional regulators in TICAE cells at the indicated times after irradiation and at the indicated doses.

Supplementary Data Sheet 2 **Irradiated TICAE cells differentially express genes controlling cell cycling and inflammation.** Gene ontology enrichment analysis was performed on differentially expressed genes in irradiated versus sham-irradiated TICAE cells (*n* = 3). Full list of the GO enrichment terms among upregulated and downregulated differentially expressed genes in TICAE cells at the indicated times after irradiation and at the indicated doses.

## References

[B1] Agarwal M. L. Agarwal A. Taylor W. R. Stark G. R. (1995). p53 controls both the G2/M and the G1 cell cycle checkpoints and mediates reversible growth arrest in human fibroblasts. Proc. Natl. Acad. Sci. U.S.A. 92, 8493–8497. 10.1073/pnas.92.18.8493 7667317 PMC41183

[B2] Aviner R. Shenoy A. Elroy-Stein O. Geiger T. (2015). Uncovering hidden layers of cell cycle regulation through integrative multi-omic analysis. PLoS Genet. 11:e1005554. 10.1371/journal.pgen.1005554 26439921 PMC4595013

[B3] Banáth J. P. Klokov D. MacPhail S. H. Banuelos C. A. Olive P. L. (2010). Residual gammaH2AX foci as an indication of lethal DNA lesions. BMC Cancer 10:4. 10.1186/1471-2407-10-4 20051134 PMC2819996

[B4] Barish G. D. Atkins A. R. Downes M. Olson P. Chong L. W. Nelson M.. (2008). PPARdelta regulates multiple proinflammatory pathways to suppress atherosclerosis. Proc. Natl. Acad. Sci. U.S.A. 105, 4271–4276. 10.1073/pnas.0711875105 18337509 PMC2393796

[B5] Beck R. E. Kim L. Yue N. J. Haffty B. G. Khan A. J. Goyal S. (2014). Treatment techniques to reduce cardiac irradiation for breast cancer patients treated with breast-conserving surgery and radiation therapy: a review. Front. Oncol. 4:327. 10.3389/fonc.2014.00327 25452938 PMC4231838

[B6] Benjamini Y. Hochberg Y. (1995). Controlling the false discovery rate - a practical and powerful approach to multiple testing. J. R. Stat. Soc. Ser. B Methodol. 57, 289–300.

[B7] Bernhard E. J. Maity A. Muschel R. J. McKenna W. G. (1995). Effects of ionizing radiation on cell cycle progression. Rev. Radiat. Environ. Biophys. 34, 79–83. 10.1007/BF01275210 7652155

[B8] Bertoli C. Skotheim J. M. de Bruin R. A. (2013). Control of cell cycle transcription during G1 and S phases. Nat. Rev. Mol. Cell Biol. 14, 518–528. 10.1038/nrm3629 23877564 PMC4569015

[B9] Bhattacharya S. Asaithamby A. (2016). Ionizing radiation and heart risks. Semin. Cell Dev. Biol. 58, 14–25. 10.1016/j.semcdb.2016.01.045 26849909

[B10] Bolanos-Garcia V. M. Blundell T. L. (2011). BUB1 and BUBR1: multifaceted kinases of the cell cycle. Trends Biochem. Sci. 36, 141–150. 10.1016/j.tibs.2010.08.004 20888775 PMC3061984

[B11] Borghini A. Gianicolo E. A. Picano E. Andreassi M. G. (2013). Ionizing radiation and atherosclerosis: current knowledge and future challenges. Atherosclerosis 230, 40–47. 10.1016/j.atherosclerosis.2013.06.010 23958250

[B12] Boring L. Gosling J. Cleary M. Charo I. F. (1998). Decreased lesion formation in CCR2-/- mice reveals a role for chemokines in the initiation of atherosclerosis. Nature 394, 894–897. 10.1038/29788 9732872

[B13] Chen E. Y. Tan C. M. Kou Y. Duan Q. Wang Z. Meirelles G. V.. (2013). Enrichr: interactive and collaborative HTML5 gene list enrichment analysis tool. BMC Bioinformatics 14:128. 10.1186/1471-2105-14-128 23586463 PMC3637064

[B14] Collins R. G. Velji R. Guevara N. V. Hicks M. J. Chan L. Beaudet A. L. (2000). P-Selectin or intercellular adhesion molecule (ICAM)-1 deficiency substantially protects against atherosclerosis in apolipoprotein E-deficient mice. J. Exp. Med. 191, 189–194. 10.1084/jem.191.1.189 10620617 PMC2195808

[B15] Darby S. C. Cutter D. J. Boerma M. Constine L. S. Fajardo L. F. Kodama K.. (2010). Radiation-related heart disease: current knowledge and future prospects. Int. J. Radiat. Oncol. Biol. Phys. 76, 656–665. 10.1016/j.ijrobp.2009.09.064 20159360 PMC3910096

[B16] Darby S. C. Ewertz M. McGale P. Bennet A. M. Blom-Goldman U. Bronnum D.. (2013). Risk of ischemic heart disease in women after radiotherapy for breast cancer. N. Engl. J. Med. 368, 987–998. 10.1056/NEJMoa1209825 23484825

[B17] Derynck R. Zhang Y. E. (2003). Smad-depenent and Smad-independent pathways in TGF-beta family signalling. Nature 425, 577–584. 10.1038/nature02006 14534577

[B18] De Vos W. H. Van Neste L. Dieriks B. Joss G. H. Van Oostveldt P. (2010). High content image cytometry in the context of subnuclear organization. Cytometry A 77, 64–75. 10.1002/cyto.a.20807 19821512

[B19] Dimri G. P. Lee X. Basile G. Acosta M. Scott G. Roskelley C.. (1995). A biomarker that identifies senescent human cells in culture and in aging skin *in vivo*. Proc. Natl. Acad. Sci. U.S.A. 92, 9363–9367. 10.1073/pnas.92.20.9363 7568133 PMC40985

[B20] Eden E. Lipson D. Yogev S. Yakhini Z. (2007). Discovering motifs in ranked lists of DNA sequences. PLoS Comput. Biol. 3:e39. 10.1371/journal.pcbi.0030039 17381235 PMC1829477

[B21] Eden E. Navon R. Steinfeld I. Lipson D. Yakhini Z. (2009). GOrilla: a tool for discovery and visualization of enriched GO terms in ranked gene lists. BMC Bioinformatics 10:48. 10.1186/1471-2105-10-48 19192299 PMC2644678

[B22] Evangelou K. Lougiakis N. Rizou S. V. Kotsinas A. Kletsas D. Muñoz-Espín D.. (2017). Robust, universal biomarker assay to detect senescent cells in biological specimens. Aging Cell 16, 192–197. 10.1111/acel.12545 28165661 PMC5242262

[B23] Fazel R. Krumholz H. M. Wang Y. Ross J. S. Chen J. Ting H. H.. (2009). Exposure to low-dose ionizing radiation from medical imaging procedures. N. Engl. J. Med. 361, 849–857. 10.1056/NEJMoa0901249 19710483 PMC3707303

[B24] Fiuza C. Bustin M. Talwar S. Tropea M. Gerstenberger E. Shelhamer J. H.. (2003). Inflammation-promoting activity of HMGB1 on human microvascular endothelial cells. Blood 101, 2652–2660. 10.1182/blood-2002-05-1300 12456506

[B25] Freund A. Orjalo A. V. Desprez P. Y. Campisi J. (2010). Inflammatory networks during cellular senescence: causes and consequences. Trends Mol. Med. 16, 238–246. 10.1016/j.molmed.2010.03.003 20444648 PMC2879478

[B26] Gallo R. L. Dorschner R. A. Takashima S. Klagsbrun M. Eriksson E. Bernfield M. (1997). Endothelial cell surface alkaline phosphatase activity is induced by IL-6 released during wound repair. J. Invest. Dermatol. 109, 597–603. 10.1111/1523-1747.ep12337529 9326397

[B27] Georgakilas A. G. Pavlopoulou A. Louka M. Nikitaki Z. Vorgias C. E. Bagos P. G.. (2015). Emerging molecular networks common in ionizing radiation, immune and inflammatory responses by employing bioinformatics approaches. Cancer Lett. 368, 164–172. 10.1016/j.canlet.2015.03.021 25841996

[B28] Gerdes J. Schwab U. Lemke H. Stein H. (1983). Production of a mouse monoclonal antibody reactive with a human nuclear antigen associated with cell proliferation. Int. J. Cancer 31, 13–20. 10.1002/ijc.2910310104 6339421

[B29] Gottfried K.-L. D. Penn G. U. S. Nuclear Regulatory Commission (1996). Medical Use Program., and Institute of Medicine (U.S.). Committee for Review and Evaluation of the Medical Use Program of the Nuclear Regulatory Commission. Radiation in Medicine : a Need for Regulatory Reform. Washington, DC: National Academy Press.

[B30] Hallahan D. E. Virudachalam S. (1997a). Intercellular adhesion molecule 1 knockout abrogates radiation induced pulmonary inflammation. Proc. Natl. Acad. Sci. U.S.A. 94, 6432–6437. 10.1073/pnas.94.12.6432 9177235 PMC21067

[B31] Hallahan D. E. Virudachalam S. (1997b). Ionizing radiation mediates expression of cell adhesion molecules in distinct histological patterns within the lung. Cancer Res. 57, 2096–2099. 9187101

[B32] Harrington J. R. (2000). The role of MCP-1 in atherosclerosis. Stem Cells 18, 65–66. 10.1634/stemcells.18-1-65 10661575

[B33] Haubner F. Leyh M. Ohmann E. Pohl F. Prantl L. Gassner H. G. (2013). Effects of external radiation in a co-culture model of endothelial cells and adipose-derived stem cells. Radiat. Oncol. 8:66. 10.1186/1748-717X-8-66 23514369 PMC3653709

[B34] Hildebrandt G. (2010). Non-cancer diseases and non-targeted effects. Mutat. Res. 687, 73–77. 10.1016/j.mrfmmm.2010.01.007 20097211

[B35] Hingorani A. D. Cross J. Kharbanda R. K. Mullen M. J. Bhagat K. Taylor M.. (2000). Acute systemic inflammation impairs endothelium-dependent dilatation in humans. Circulation 102, 994–999. 10.1161/01.CIR.102.9.994 10961963

[B36] Hirase T. Node K. (2012). Endothelial dysfunction as a cellular mechanism for vascular failure. Am. J. Physiol. Heart Circ. Physiol. 302, H499–H505. 10.1152/ajpheart.00325.2011 22081698

[B37] Hoving S. Heeneman S. Gijbels M. J. Te Poele J. A. Visser N. Cleutjens J.. (2012). Irradiation induces different inflammatory and thrombotic responses in carotid arteries of wildtype C57BL/6J and atherosclerosis-prone ApoE(-/-) mice. Radiother. Oncol. 105, 365–370. 10.1016/j.radonc.2012.11.001 23245647

[B38] Kempe S. Kestler H. Lasar A. Wirth T. (2005). NF-kappaB controls the global pro-inflammatory response in endothelial cells: evidence for the regulation of a pro-atherogenic program. Nucleic Acids Res. 33, 5308–5319. 10.1093/nar/gki836 16177180 PMC1226313

[B39] Kleiner R. E. Verma P. Molloy K. R. Chait B. T. Kapoor T. M. (2015). Chemical proteomics reveals a gammaH2AX-53BP1 interaction in the DNA damage response. Nat. Chem. Biol. 11, 807–814. 10.1038/nchembio.1908 26344695 PMC4589150

[B40] Kojima H. Inoue T. Kunimoto H. Nakajima K. (2013). IL-6-STAT3 signaling and premature senescence. JAKSTAT 2:e25763. 10.4161/jkst.25763 24416650 PMC3876432

[B41] Kuleshov M. V. Jones M. R. Rouillard A. D. Fernandez N. F. Duan Q. Wang Z.. (2016). Enrichr: a comprehensive gene set enrichment analysis web server 2016 update. Nucleic Acids Res. 44, W90–W97. 10.1093/nar/gkw377 27141961 PMC4987924

[B42] Lane D. P. (1992). Cancer. p53, guardian of the genome. Nature 358, 15–16. 10.1038/358015a0 1614522

[B43] Libby P. (2002). Inflammation in atherosclerosis. Nature 420, 868–874. 10.1038/nature01323 12490960

[B44] Libby P. Ridker P. M. Hansson G. K. (2011). Progress and challenges in translating the biology of atherosclerosis. Nature 473, 317–325. 10.1038/nature10146 21593864

[B45] Little M. P. Tawn E. J. Tzoulaki I. Wakeford R. Hildebrandt G. Paris F.. (2008). A systematic review of epidemiological associations between low and moderate doses of ionizing radiation and late cardiovascular effects, and their possible mechanisms. Radiat. Res. 169, 99–109. 10.1667/RR1070.1 18159955

[B46] Lowe D. Raj K. (2014). Premature aging induced by radiation exhibits pro-atherosclerotic effects mediated by epigenetic activation of CD44 expression. Aging Cell 13, 900–910. 10.1111/acel.12253 25059316 PMC4331742

[B47] Lusis A. J. (2000). Atherosclerosis. Nature 407, 233–241. 10.1093/nar/29.9.e45 11001066 PMC2826222

[B48] MacDonald S. M. Jimenez R. Paetzold P. Adams J. Beatty J. DeLaney T. F.. (2013). Proton radiotherapy for chest wall and regional lymphatic radiation; dose comparisons and treatment delivery. Radiat. Oncol. 8:71. 10.1186/1748-717X-8-71 23521809 PMC3627609

[B49] Magné N. Toillon R. A. Bottero V. Didelot C. Houtte P. V. Gerard J. P.. (2006). NF-kappaB modulation and ionizing radiation: mechanisms and future directions for cancer treatment. Cancer Lett. 231, 158–168. 10.1016/j.canlet.2005.01.022 16399220

[B50] Minamino T. Miyauchi H. Yoshida T. Ishida Y. Yoshida H. Komuro I. (2002). Endothelial cell senescence in human atherosclerosis: role of telomere in endothelial dysfunction. Circulation 105, 1541–1544. 10.1161/01.CIR.0000013836.85741.17 11927518

[B51] Morey J. S. Ryan J. C. Van Dolah F. M. (2006). Microarray validation: factors influencing correlation between oligonucleotide microarrays and real-time PCR. Biol. Proced. Online 8, 175–193. 10.1251/bpo126 17242735 PMC1779618

[B52] Nikitaki Z. Nikolov V. Mavragani I. V. Mladenov E. Mangelis A. Laskaratou D. A.. (2016). Measurement of complex DNA damage induction and repair in human cellular systems after exposure to ionizing radiations of varying linear energy transfer (LET). Free Radic. Res. 50(Suppl 1), S64–S78. 10.1080/10715762.2016.1232484 27593437

[B53] Ngwa W. Kumar R. Sridhar S. Korideck H. Zygmanski P. Cormack R. A.. (2014). Targeted radiotherapy with gold nanoparticles: current status and future perspectives. Nanomedicine 9, 1063–1082. 10.2217/nnm.14.55 24978464 PMC4143893

[B54] Paoletti R. Gotto A. M. Jr. Hajjar D. P. (2004). Inflammation in atherosclerosis and implications for therapy. Circulation 109(23 Suppl. 1), III20–26. 10.1161/01.cir.0000131514.71167.2e 15198962

[B55] Pfaffl M. W. (2001). A new mathematical model for relative quantification in real-time RT-PCR. Nucleic Acids Res. 29:45. 10.1093/nar/29.9.e45 11328886 PMC55695

[B56] Piqueras L. Reynolds A. R. Hodivala-Dilke K. M. Alfranca A. Redondo J. M. Hatae T.. (2007). Activation of PPARbeta/delta induces endothelial cell proliferation and angiogenesis. Arterioscler. Thromb. Vasc. Biol. 27, 63–69. 10.1161/01.ATV.0000250972.83623.61 17068288

[B57] Raviraj J. Bokkasam V. K. Kumar V. S. Reddy U. S. Suman V. (2014). Radiosensitizers, radioprotectors, and radiation mitigators. Indian J. Dent. Res. 25, 83–90. 10.4103/0970-9290.131142 24748306

[B58] Rombouts C. Aerts A. Quintens R. Baselet B. El-Saghire H. Harms-Ringdahl M.. (2014). Transcriptomic profiling suggests a role for IGFBP5 in premature senescence of endothelial cells after chronic low dose rate irradiation. Int. J. Radiat. Biol. 90, 560–574. 10.3109/09553002.2014.905724 24646080

[B59] Rufini A. Tucci P. Celardo I. Melino G. (2013). Senescence and aging: the critical roles of p53. Oncogene 32, 5129–5143. 10.1038/onc.2012.640 23416979

[B60] Schieffer B. Selle T. Hilfiker A. Hilfiker-Kleiner D. Grote K. Tietge U. J.. (2004). Impact of interleukin-6 on plaque development and morphology in experimental atherosclerosis. Circulation 110, 3493–3500. 10.1161/01.CIR.0000148135.08582.97 15557373

[B61] Schindelin J. Arganda-Carreras I. Frise E. Kaynig V. Longair M. Pietzsch T.. (2012). Fiji: an open-source platform for biological-image analysis. Nat. Methods 9, 676–682. 10.1038/nmeth.2019 22743772 PMC3855844

[B62] Schuett H. Luchtefeld M. Grothusen C. Grote K. Schieffer B. (2009). How much is too much? Interleukin-6 and its signalling in atherosclerosis. Thromb. Haemost. 102, 215–222. 10.1160/th09-05-0297 19652871

[B63] Shimizu Y. Kodama K. Nishi N. Kasagi F. Suyama A. Soda M.. (2010). Radiation exposure and circulatory disease risk: Hiroshima and Nagasaki atomic bomb survivor data. 1950–2003. BMJ 340:b5349. 10.1136/bmj.b5349 20075151 PMC2806940

[B64] Stewart F. A. Heeneman S. Te Poele J. Kruse J. Russell N. S. Gijbels M. (2006). Ionizing radiation accelerates the development of atherosclerotic lesions in ApoE^−/−^ mice and predisposes to an inflammatory plaque phenotype prone to hemorrhage. Am. J. Pathol. 168, 649–658. 10.2353/ajpath.2006.050409 16436678 PMC1606487

[B65] Sun W. Jiao Y. Cui B. Gao X. Xia Y. Zhao Y. (2013). Immune complexes activate human endothelium involving the cell-signaling HMGB1-RAGE axis in the pathogenesis of lupus vasculitis. Lab. Invest. 93, 626–638. 10.1038/labinvest.2013.61 23628898

[B66] Supek F. Bošnjak M. Škunca N. Šmuc T. (2011). REVIGO summarizes and visualizes long lists of gene ontology terms. PLoS ONE 6:e21800. 10.1371/journal.pone.0021800 21789182 PMC3138752

[B67] Uberti F. Lattuada D. Morsanuto V. Nava U. Bolis G. Vacca G.. (2014). Vitamin D protects human endothelial cells from oxidative stress through the autophagic and survival pathways. J. Clin. Endocrinol. Metab. 99, 1367–1374. 10.1210/jc.2013-2103 24285680

[B68] UNSCEAR (2008). Sources and Effects of Ionizing Radiation. Annex A: Medical Radiation Exposures.

[B69] Van Der Meeren A. Squiban C. Gourmelon P. Lafont H. Gaugler M. H. (1999). Differential regulation by IL-4 and IL-10 of radiation-induced IL-6 and IL-8 production and ICAM-1 expression by human endothelial cells. Cytokine 11, 831–838. 10.1006/cyto.1999.0497 10547270

[B70] Verreet T. Quintens R. Van Dam D. Verslegers M. Tanori M. Casciati A.. (2015). A multidisciplinary approach unravels early and persistent effects of X-ray exposure at the onset of prenatal neurogenesis. J. Neurodev. Disord. 7:3. 10.1186/1866-1955-7-3 26029273 PMC4448911

[B71] Vita J. A. Keaney J. F. Jr. (2002). Endothelial function: a barometer for cardiovascular risk? Circulation 106, 640–642. 10.1161/01.CIR.0000028581.07992.56 12163419

[B72] Wajapeyee N. Serra R. W. Zhu X. Mahalingam M. Green M. R. (2008). Oncogenic BRAF induces senescence and apoptosis through pathways mediated by the secreted protein IGFBP7. Cell 132, 363–374. 10.1016/j.cell.2007.12.032 18267069 PMC2266096

[B73] Wang H. Adhikari S. Butler B. E. Pandita T. K. Mitra S. Hegde M. L. (2014). A perspective on chromosomal double strand break markers in mammalian cells. Jacobs J. Radiat. Oncol. 1:003. 25614903 PMC4299656

[B74] Wang X. Liu J. Jiang L. Wei X. Niu C. Wang R.. (2016a). Bach1 Induces Endothelial Cell Apoptosis and Cell-Cycle Arrest through ROS Generation. Oxid. Med. Cell. Longev. 2016:6234043. 10.1155/2016/6234043 27057283 PMC4789484

[B75] Wang Y. Boerma M. Zhou D. (2016b). Ionizing Radiation-Induced endothelial cell senescence and cardiovascular diseases. Radiat. Res. 186, 153–161. 10.1667/RR14445.1 27387862 PMC4997805

[B76] Wasylishen A. R. Penn L. Z. (2010). Myc: the beauty and the beast. Genes Cancer 1, 532–541. 10.1177/1947601910378024 21779457 PMC3092215

[B77] Weber C. Noels H. (2011). Atherosclerosis: current pathogenesis and therapeutic options. Nat. Med. 17, 1410–1422. 10.1038/nm.2538 22064431

[B78] Widmer R. J. Lerman A. (2014). Endothelial dysfunction and cardiovascular disease. Glob. Cardiol. Sci. Pract. 2014, 291–308. 10.5339/gcsp.2014.43 25780786 PMC4352682

[B79] Yentrapalli R. Azimzadeh O. Barjaktarovic Z. Sarioglu H. Wojcik A. Harms-Ringdahl M.. (2013a). Quantitative proteomic analysis reveals induction of premature senescence in human umbilical vein endothelial cells exposed to chronic low-dose rate gamma radiation. Proteomics 13, 1096–1107. 10.1002/pmic.201200463 23349028

[B80] Yentrapalli R. Azimzadeh O. Sriharshan A. Malinowsky K. Merl J. Wojcik A.. (2013b). The PI3K/Akt/mTOR pathway is implicated in the premature senescence of primary human endothelial cells exposed to chronic radiation. PLoS ONE 8:e70024. 10.1371/journal.pone.0070024 23936371 PMC3731291

[B81] Yu H. (2012). Typical cell signaling response to ionizing radiation: DNA damage and extranuclear damage. Chin. J. Cancer Res. 24, 83–89. 10.1007/s11670-012-0083-1 23357898 PMC3555267

[B82] Zona S. Bella L. Burton M. J. Nestal de Moraes G. Lam E. W. (2014). FOXM1: an emerging master regulator of DNA damage response and genotoxic agent resistance. Biochim. Biophys. Acta 1839, 1316–1322. 10.1016/j.bbagrm.2014.09.016 25287128 PMC4316173

