# Supplementary material for: Functional Gene Analysis Reveals Cell Cycle Changes and Inflammation in Endothelial Cells Irradiated with a Single X-ray Dose
Source: Front Pharmacol. 2017 Apr 25;8:213. doi: 10.3389/fphar.2017.00213 (PMC5404649; doi:10.3389/fphar.2017.00213)
Supplement: Supplementary file 5 [file DataSheet5.DOCX]

Supplementary Material

**Functional Gene Analysis Reveals Cell Cycle Changes and Inflammation in Endothelial Cells Irradiated with a Single X-ray Dose**

**Bjorn Baselet^1,2^, Niels Belmans^1,3^, Emma Coninx^1^, Donna Lowe^4^, Ann Janssen^1^, Arlette Michaux^1^, Kevin Tabury^1,5^, Kenneth Raj^4^, Roel Quintens^1^, Abderrafi Mohammed Benotmane^1,$^, Sarah Baatout^1,6,$^, Pierre Sonveaux^2,$^ An Aerts^1,^** $**^,*^**

*** Correspondence:** An Aerts: [an.aerts@sckcen.be](mailto:an.aerts@sckcen.be)


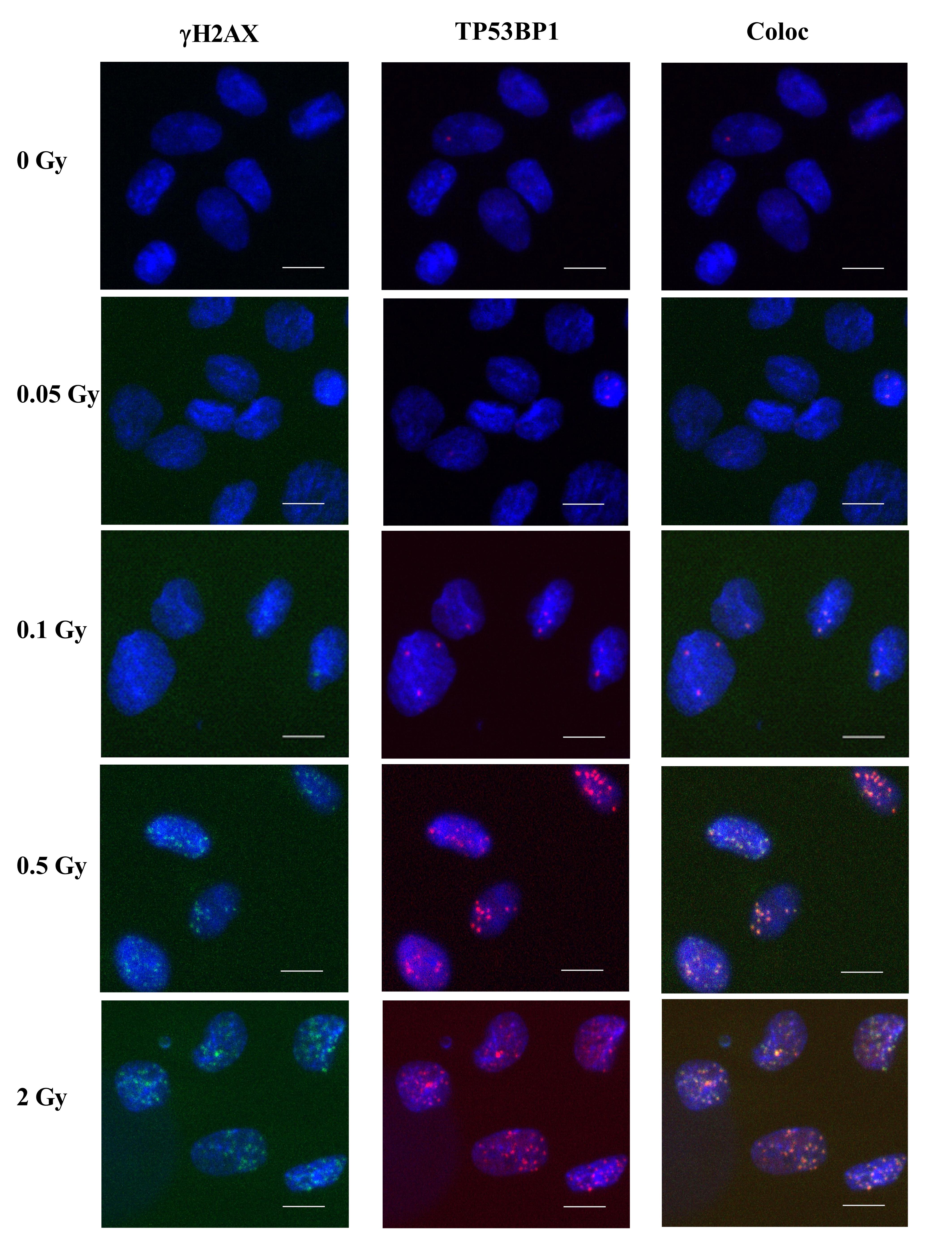


**Supplementary Figure 3.** Representative images showing γH2AX (green), TP53BP1 (red) and γH2AX+TP53BP1 (yellow) foci in DAPI stained nuclei (blue) of TICAE cells 1 hour after irradiation with a single X-ray dose of either 0, 0.05, 0.1, 0.5 or 2 Gy. Scale bar, 10 µm
