# Supplementary material for: Functional Gene Analysis Reveals Cell Cycle Changes and Inflammation in Endothelial Cells Irradiated with a Single X-ray Dose
Source: Front Pharmacol. 2017 Apr 25;8:213. doi: 10.3389/fphar.2017.00213 (PMC5404649; doi:10.3389/fphar.2017.00213)
Supplement: Supplementary file 8 [file Table1.DOCX]

Supplementary Material

**Functional Gene Analysis Reveals Cell Cycle Changes and Inflammation in Endothelial Cells Irradiated with a Single X-ray Dose**

**Bjorn Baselet^1,2^, Niels Belmans^1,3^, Emma Coninx^1^, Donna Lowe^4^, Ann Janssen^1^, Arlette Michaux^1^, Kevin Tabury^1,5^, Kenneth Raj^4^, Roel Quintens^1^, Abderrafi Mohammed Benotmane^1,$^, Sarah Baatout^1,6,$^, Pierre Sonveaux^2,$^ An Aerts^1,^** $**^,*^**

*** Correspondence:** An Aerts: [an.aerts@sckcen.be](mailto:an.aerts@sckcen.be)

**Supplementary table 1. Differentially expressed genes in TICAE cells irradiated with a single X-ray dose of 2 Gy.***

| Day 1 | | | Day 7 | | | Day 14 | | |
| --- | --- | --- | --- | --- | --- | --- | --- | --- |
| 2 Gy vs. 0 Gy | | | 2 Gy vs. 0 Gy | | | 2 Gy vs. 0 Gy | | |
| Probeset ID | Gene symbol | Fold change | Probeset ID | Gene symbol | Fold change | Probeset ID | Gene symbol | Fold change |
| 16767115 | RPSAP52 | 3.01 | 16698947 | RNU5A-8P | 8.96 | 16787362 | RN7SKP255 | 3.60 |
| 16735751 | LYVE1 | 2.96 | 17022133 | LINC00577 | 4.01 | 16992789 | RP11-843P14.2 | 3.18 |
| 17039299 | HLA-B | 2.77 | 17100711 |  | 3.65 | 16997676 | MTRNR2L2 | 2.73 |
| 16753853 | MDM2 | 2.73 | 16787362 | RN7SKP255 | 2.59 | 16744154 | RP11-25I9.2 | 2.42 |
| 17022133 | LINC00577 | 2.60 | 16876431 |  | 2.48 | 16818543 |  | 2.38 |
| 17005167 | RNU6-190P | 2.58 | 16782050 | MGC40069 | 2.42 | 17074080 |  | 2.33 |
| 16988913 | RNU6ATAC10P | 2.50 | 16775583 | RNY3P7 | 2.39 | 16782050 | MGC40069 | 2.31 |
| 16744154 | RP11-25I9.2 | 2.49 | 16988913 | RNU6ATAC10P | 2.36 | 17005167 | RNU6-190P | 2.31 |
| 16966279 | RNA5SP160 | 2.41 | 16735751 | LYVE1 | 2.33 | 16974115 |  | 2.23 |
| 16831787 |  | 2.35 | 16974115 |  | 2.30 | 16776021 | RNU6-83P | 2.23 |
| 16787362 | RN7SKP255 | 2.34 | 16976012 |  | 2.18 | 16988913 | RNU6ATAC10P | 2.06 |
| 16974115 |  | 2.33 | 16660734 | MIR378F | 2.16 | 16996800 |  | 2.03 |
| 16823889 | MIR548H2 | 2.32 | 17051553 | CPA4 | 2.11 | 16900737 |  | 2.01 |
| 16998510 |  | 2.26 | 16831787 |  | 2.08 | 17094586 |  | 2.00 |
| 16726880 | NEAT1 | 2.20 | 17005167 | RNU6-190P | 2.05 | 16862604 | CD79A | 1.94 |
| 16776616 | SOX1 | 2.18 | 16721566 | RP11-324J3.1 | 2.03 | 16697654 | MIR181A1HG | 1.86 |
| 17014959 | LOC102723922 | 2.15 | 17016383 | HIST1H4D | 1.98 | 16833917 | LRRC3C | 1.84 |
| 17074080 |  | 2.10 | 16698714 |  | 1.94 | 16920528 |  | 1.84 |
| 16848055 |  | 2.10 | 16967875 | PARM1 | 1.91 | 16921827 | MIR155HG | 1.83 |
| 16696425 | TNFSF4 | 2.08 | 16774053 | CCNA1 | 1.90 | 16991667 |  | 1.82 |
| 16660734 | MIR378F | 2.05 | 17074080 |  | 1.90 | 16819792 | RNA5SP428 | 1.81 |
| 16880254 | RNU6-634P | 2.01 | 16744154 | RP11-25I9.2 | 1.89 | 17063005 | PLXNA4 | 1.81 |
| 16897446 | RNU6-439P | 1.97 | 17059119 | SEMA3C | 1.87 | 16900709 |  | 1.79 |
| 16858310 | MIR4748 | 1.96 | 16917849 | THBD | 1.85 | 16719786 |  | 1.78 |
| 16966685 | SPATA18 | 1.94 | 17043843 | TSPAN13 | 1.85 | 16734793 | OR51E2 | 1.70 |
| 16983742 | RNU6-760P | 1.92 | 16875763 | UBE2S | 1.84 | 16686037 | RP5-994D16.9 | 1.69 |
| 16877451 |  | 1.91 | 16776021 | RNU6-83P | 1.84 | 16785151 | RNU6-1162P | 1.64 |
| 16782050 | MGC40069 | 1.91 | 16903140 | CXCR4 | 1.81 | 17061532 | SYPL1 | 1.64 |
| 16997676 | MTRNR2L2 | 1.91 | 16904425 | GRB14 | 1.79 | 16722162 | PARVA | 1.64 |
| 16996956 |  | 1.90 | 17076726 | PLAT | 1.79 | 16681884 | HNRNPCL2 | 1.64 |
| 16829505 |  | 1.90 | 16966855 | KIT | 1.79 | 16782102 | TRAJ10 | 1.62 |
| 16701877 | PITRM1-AS1 | 1.90 | 17087758 | NIPSNAP3A | 1.78 | 17027679 | DPCR1 | 1.62 |
| 17042857 | AC091729.8 | 1.87 | 16660713 |  | 1.77 | 16908112 | AC012668.3 | 1.62 |
| 17073066 | RNA5SP278 | 1.86 | 16842219 |  | 1.76 | 16796406 |  | 1.57 |
| 16776021 | RNU6-83P | 1.85 | 16979985 | MGARP | 1.76 | 16828833 |  | 1.57 |
| 17047918 | CROT | 1.85 | 16702172 |  | 1.76 | 16856172 | RNA5SP462 | 1.57 |
| 17084164 | RP11-298E2.2 | 1.85 | 17027679 | DPCR1 | 1.76 | 16943241 | COL8A1 | 1.56 |
| 16784135 | RNA5SP385 | 1.83 | 16997676 | MTRNR2L2 | 1.76 | 16827041 | CDH11 | 1.55 |
| 16721371 |  | 1.83 | 16788630 | SNHG24 | 1.75 | 16819736 |  | 1.53 |
| 17072723 | RNU1-106P | 1.82 | 16884301 | AC123886.2 | 1.73 | 16897446 | RNU6-439P | 1.53 |
| 16783494 | SLC25A21-AS1 | 1.82 | 17010173 | RNU6-411P | 1.72 | 16682175 | SPATA21 | 1.52 |
| 16828833 |  | 1.81 | 16897159 | SIX2 | 1.72 | 16849623 | MIR4739 | -1.50 |
| 16661811 |  | 1.80 | 16833204 | CCL2 | 1.71 | 16684579 | MIR4254 | -1.53 |
| 16683925 | SLC9A1 | 1.80 | 16961637 | NCEH1 | 1.70 | 16952769 | CDCP1 | -1.55 |
| 16681451 | RP3-510D11.1 | 1.79 | 16993385 |  | 1.69 | 16963428 | MFI2 | -1.56 |
| 16894673 | AC008278.3 | 1.78 | 16930021 | MIR4534 | 1.67 | 16875968 |  | -1.58 |
| 16993385 |  | 1.77 | 16828833 |  | 1.66 | 16979900 |  | -1.60 |
| 16681370 | ENO1 | 1.76 | 16988423 | PRR16 | 1.64 | 16988984 | PDLIM4 | -1.60 |
| 16956613 |  | 1.73 | 17100771 |  | 1.64 | 17027504 | MIR877 | -1.61 |
| 16707923 |  | 1.72 | 16726880 | NEAT1 | 1.63 | 16907621 | MIR3130-1 | -1.61 |
| 16660309 | RP3-340N1.2 | 1.72 | 16824564 |  | 1.63 | 16668572 | CYMP | -1.61 |
| 16819792 | RNA5SP428 | 1.72 | 16855600 | CCBE1 | 1.63 | 16684080 | IFI6 | -1.63 |
| 17027679 | DPCR1 | 1.72 | 17045838 | IGFBP1 | 1.63 | 16780885 | FAM155A | -1.63 |
| 16738933 | VWCE | 1.71 | 17081106 | GSDMC | 1.63 | 17016366 | HIST1H2AB | -1.65 |
| 16683290 | ZNF436 | 1.71 | 16696425 | TNFSF4 | 1.62 | 16925461 | AP000696.2 | -1.67 |
| 17081071 | RNU4-25P | 1.70 | 16702571 | MCM10 | 1.62 | 16941998 | SPATA12 | -1.70 |
| 16991527 | CYFIP2 | 1.69 | 16684080 | IFI6 | 1.62 | 16731396 | RP11-159N11.3 | -1.88 |
| 16963113 | APOD | 1.68 | 17016366 | HIST1H2AB | 1.60 | 16924097 |  | -2.01 |
| 16928988 | TCN2 | 1.67 | 16719786 |  | 1.60 | 16989408 | MIR4461 | -2.23 |
| 16833917 | LRRC3C | 1.67 | 16833297 | FNDC8 | 1.60 |  |  |  |
| 16700218 | TRIM17 | 1.66 | 16857258 | UHRF1 | 1.59 |  |  |  |
| 17040387 | HLA-A | 1.66 | 16905436 | LINC01116 | 1.59 |  |  |  |
| 16923805 | PCBP3 | 1.65 | 16996612 | CTC-436P18.3 | 1.59 |  |  |  |
| 17084959 |  | 1.65 | 16762837 | CAPRIN2 | 1.58 |  |  |  |
| 16819736 |  | 1.65 | 16725041 | FAM111B | 1.57 |  |  |  |
| 17117604 | GAS6-AS1 | 1.65 | 17110932 |  | 1.57 |  |  |  |
| 16967875 | PARM1 | 1.65 | 16698023 | UBE2T | 1.56 |  |  |  |
| 17114007 | APLN | 1.64 | 17106067 | VSIG1 | 1.55 |  |  |  |
| 16797340 | IGHE | 1.64 | 16919962 | SULF2 | 1.54 |  |  |  |
| 16856172 | RNA5SP462 | 1.64 | 16883690 | IL1RL1 | 1.53 |  |  |  |
| 16734339 | MIR4298 | 1.64 | 17080749 | ATAD2 | 1.53 |  |  |  |
| 16719786 |  | 1.64 | 16686834 |  | 1.52 |  |  |  |
| 17085196 | ANKRD20A3 | 1.63 | 16686037 | RP5-994D16.9 | 1.52 |  |  |  |
| 16788725 | MIR329-1 | 1.62 | 16861641 | C19orf33 | 1.51 |  |  |  |
| 16797444 |  | 1.62 | 16757324 | OAS1 | 1.51 |  |  |  |
| 16698714 |  | 1.60 | 16819213 | MT1L | 1.51 |  |  |  |
| 17018720 | TMEM217 | 1.60 | 16834056 | CDC6 | 1.51 |  |  |  |
| 16665932 | GADD45A | 1.60 | 16779546 | DIAPH3 | 1.50 |  |  |  |
| 16920528 |  | 1.59 | 16868838 | SPC24 | 1.50 |  |  |  |
| 16921827 | MIR155HG | 1.59 | 16878416 | BRE | -1.50 |  |  |  |
| 16774303 | RGCC | 1.59 | 16933044 | ZNF70 | -1.50 |  |  |  |
| 16876497 |  | 1.58 | 17005094 | GMPR | -1.51 |  |  |  |
| 16824564 |  | 1.58 | 17067496 | CTD-2647L4.4 | -1.51 |  |  |  |
| 17079317 | TP53INP1 | 1.58 | 16915245 | APCDD1L-AS1 | -1.52 |  |  |  |
| 16919962 | SULF2 | 1.58 | 16744205 | ARHGAP20 | -1.52 |  |  |  |
| 16991667 |  | 1.58 | 16934780 |  | -1.52 |  |  |  |
| 17014680 | RP11-568A7.2 | 1.57 | 16732807 | VWA5A | -1.52 |  |  |  |
| 16813199 | MESP1 | 1.57 | 17071144 | SDC2 | -1.52 |  |  |  |
| 16920315 | NFATC2 | 1.56 | 16995715 | LOC100506548 | -1.53 |  |  |  |
| 16996937 | RNU6-724P | 1.56 | 16685875 | SLFNL1 | -1.53 |  |  |  |
| 16991942 | RN7SKP60 | 1.56 | 16669796 | TXNIP | -1.55 |  |  |  |
| 17099685 |  | 1.56 | 16675045 | HMCN1 | -1.55 |  |  |  |
| 16769569 | NUAK1 | 1.55 | 17029003 | HLA-C | -1.55 |  |  |  |
| 16661025 | RNU6-110P | 1.55 | 16838169 | SNHG20 | -1.55 |  |  |  |
| 16953862 | QARS | 1.55 | 16756310 | TCP11L2 | -1.55 |  |  |  |
| 16721280 | TRIM22 | 1.54 | 17079248 | KIAA1429 | -1.56 |  |  |  |
| 16776491 |  | 1.54 | 17040387 | HLA-A | -1.56 |  |  |  |
| 16884301 | AC123886.2 | 1.54 | 16829426 | DBNDD1 | -1.57 |  |  |  |
| 16859795 | GDF15 | 1.54 | 16847676 | GH2 | -1.58 |  |  |  |
| 16734793 | OR51E2 | 1.54 | 16840113 | CXCL16 | -1.58 |  |  |  |
| 16860386 |  | 1.53 | 16862284 | LTBP4 | -1.59 |  |  |  |
| 16665796 | SGIP1 | 1.53 | 16952769 | CDCP1 | -1.59 |  |  |  |
| 17117542 |  | 1.52 | 16789526 | ZBTB42 | -1.60 |  |  |  |
| 17073358 | RP11-909N17.2 | 1.52 | 17088760 | PTGS1 | -1.60 |  |  |  |
| 16864920 | FAM90A27P | 1.51 | 17059955 | PDK4 | -1.61 |  |  |  |
| 17075014 | MTUS1 | 1.51 | 16957396 | CCDC80 | -1.61 |  |  |  |
| 16911139 | PRND | 1.51 | 16852573 | NEDD4L | -1.62 |  |  |  |
| 16830914 | PFAS | -1.50 | 16959386 | SLCO2A1 | -1.62 |  |  |  |
| 17087343 | NCBP1 | -1.50 | 16688615 | SLC44A5 | -1.63 |  |  |  |
| 17072313 | TBC1D31 | -1.50 | 16761820 | MGP | -1.64 |  |  |  |
| 16687464 |  | -1.51 | 16790744 | SLC7A8 | -1.65 |  |  |  |
| 17102948 | ZNF674-AS1 | -1.51 | 16933030 | GUSBP11 | -1.65 |  |  |  |
| 17117418 |  | -1.51 | 16876147 | ZNF132 | -1.66 |  |  |  |
| 17062502 | POT1 | -1.51 | 16806538 | GOLGA8R | -1.66 |  |  |  |
| 16829085 | SLC7A5 | -1.51 | 16669850 | ITGA10 | -1.67 |  |  |  |
| 16959325 | TOPBP1 | -1.51 | 17092331 | PTPRD | -1.68 |  |  |  |
| 16851397 | RBBP8 | -1.51 | 16942367 | RNA5SP134 | -1.72 |  |  |  |
| 16978995 | ELOVL6 | -1.51 | 16686640 | KNCN | -1.73 |  |  |  |
| 16871567 | ALKBH6 | -1.52 | 16675398 | CFH | -1.74 |  |  |  |
| 16852445 | C18orf54 | -1.52 | 16802022 | ZNF609 | -1.82 |  |  |  |
| 16699877 | LBR | -1.52 | 16671187 | NPR1 | -1.83 |  |  |  |
| 17057413 | SNHG15 | -1.52 | 16757178 | RP3-462E2.3 | -1.87 |  |  |  |
| 16728518 | FAM86C1 | -1.52 | 16760257 | VWF | -1.89 |  |  |  |
| 16772625 | POLE | -1.52 | 16928293 |  | -1.97 |  |  |  |
| 16952769 | CDCP1 | -1.52 | 17046284 | SUMF2 | -2.17 |  |  |  |
| 17088185 | PRPF4 | -1.52 | 16780317 | CLDN10-AS1 | -2.26 |  |  |  |
| 16732880 | SPA17 | -1.52 | 16761776 |  | -2.29 |  |  |  |
| 16988423 | PRR16 | -1.52 | 16667155 | SNORD21 | -2.37 |  |  |  |
| 16942991 | ARL13B | -1.52 | 16938133 | GALNT15 | -2.40 |  |  |  |
| 16883690 | IL1RL1 | -1.52 |  |  |  |  |  |  |
| 16877888 | EPT1 | -1.52 |  |  |  |  |  |  |
| 16831383 | ADORA2B | -1.53 |  |  |  |  |  |  |
| 16683875 | GPN2 | -1.53 |  |  |  |  |  |  |
| 16981506 | HMGB2 | -1.53 |  |  |  |  |  |  |
| 16777384 | SACS | -1.53 |  |  |  |  |  |  |
| 16722603 | LDHA | -1.53 |  |  |  |  |  |  |
| 17111594 | SPIN4 | -1.53 |  |  |  |  |  |  |
| 16663033 | SMAP2 | -1.53 |  |  |  |  |  |  |
| 16929199 | LOC54944 | -1.54 |  |  |  |  |  |  |
| 16662338 | ZMYM1 | -1.54 |  |  |  |  |  |  |
| 16799517 | KNSTRN | -1.54 |  |  |  |  |  |  |
| 17086987 | PTPDC1 | -1.54 |  |  |  |  |  |  |
| 17097914 | PHF19 | -1.54 |  |  |  |  |  |  |
| 16850477 | TYMS | -1.54 |  |  |  |  |  |  |
| 17021521 | RNGTT | -1.55 |  |  |  |  |  |  |
| 16731169 | DLAT | -1.55 |  |  |  |  |  |  |
| 16959148 | ASTE1 | -1.55 |  |  |  |  |  |  |
| 17016506 | HIST1H4L | -1.55 |  |  |  |  |  |  |
| 16661806 | RN7SKP91 | -1.55 |  |  |  |  |  |  |
| 16995705 | RPL37 | -1.55 |  |  |  |  |  |  |
| 16678496 | HIST3H2BB | -1.55 |  |  |  |  |  |  |
| 16753800 | NUP107 | -1.56 |  |  |  |  |  |  |
| 16683887 | GPATCH3 | -1.56 |  |  |  |  |  |  |
| 17117581 | LOC100128816 | -1.56 |  |  |  |  |  |  |
| 16830778 | TMEM88 | -1.56 |  |  |  |  |  |  |
| 16970231 | EXOSC9 | -1.57 |  |  |  |  |  |  |
| 16661117 | CEP85 | -1.57 |  |  |  |  |  |  |
| 16744125 | KDELC2 | -1.57 |  |  |  |  |  |  |
| 16910501 | DTYMK | -1.57 |  |  |  |  |  |  |
| 16760162 | C12orf4 | -1.57 |  |  |  |  |  |  |
| 16725049 | FAM111A | -1.57 |  |  |  |  |  |  |
| 17025693 | RP1-167A14.2 | -1.57 |  |  |  |  |  |  |
| 16848793 | SLC25A19 | -1.57 |  |  |  |  |  |  |
| 16922495 | CBR3 | -1.57 |  |  |  |  |  |  |
| 17092615 | PSIP1 | -1.58 |  |  |  |  |  |  |
| 16932914 | ZNF280B | -1.58 |  |  |  |  |  |  |
| 16748490 | APOLD1 | -1.58 |  |  |  |  |  |  |
| 16973693 | HAUS3 | -1.58 |  |  |  |  |  |  |
| 16757347 | OAS3 | -1.58 |  |  |  |  |  |  |
| 16943919 | C3orf52 | -1.58 |  |  |  |  |  |  |
| 17093724 | ARHGEF39 | -1.58 |  |  |  |  |  |  |
| 16735545 | NRIP3 | -1.59 |  |  |  |  |  |  |
| 16785631 | EIF2S1 | -1.59 |  |  |  |  |  |  |
| 16755498 | TMPO | -1.59 |  |  |  |  |  |  |
| 17051965 | NUP205 | -1.59 |  |  |  |  |  |  |
| 17024227 | REPS1 | -1.59 |  |  |  |  |  |  |
| 16664576 |  | -1.59 |  |  |  |  |  |  |
| 17005535 |  | -1.59 |  |  |  |  |  |  |
| 16909588 | TIGD1 | -1.60 |  |  |  |  |  |  |
| 17032533 | HLA-DPA1 | -1.60 |  |  |  |  |  |  |
| 16838887 | SLC25A10 | -1.60 |  |  |  |  |  |  |
| 16766719 | METTL1 | -1.60 |  |  |  |  |  |  |
| 17014309 | ACAT2 | -1.60 |  |  |  |  |  |  |
| 16877762 | CENPO | -1.61 |  |  |  |  |  |  |
| 16908985 | AP1S3 | -1.61 |  |  |  |  |  |  |
| 16747287 | NCAPD2 | -1.61 |  |  |  |  |  |  |
| 16885028 | TMEM177 | -1.61 |  |  |  |  |  |  |
| 16664118 | NASP | -1.61 |  |  |  |  |  |  |
| 16829464 |  | -1.61 |  |  |  |  |  |  |
| 17064105 | EZH2 | -1.62 |  |  |  |  |  |  |
| 16907960 | BARD1 | -1.62 |  |  |  |  |  |  |
| 16754917 | C12orf29 | -1.62 |  |  |  |  |  |  |
| 16662945 | MFSD2A | -1.62 |  |  |  |  |  |  |
| 16766137 | TIMELESS | -1.63 |  |  |  |  |  |  |
| 16853042 | TIMM21 | -1.63 |  |  |  |  |  |  |
| 16768579 | CEP83 | -1.63 |  |  |  |  |  |  |
| 17117588 | RP11-669B18.1 | -1.63 |  |  |  |  |  |  |
| 16977066 | NUP54 | -1.63 |  |  |  |  |  |  |
| 16922584 | CHAF1B | -1.63 |  |  |  |  |  |  |
| 16781482 | PARP2 | -1.63 |  |  |  |  |  |  |
| 17086353 | RMI1 | -1.63 |  |  |  |  |  |  |
| 16996645 | DIMT1 | -1.63 |  |  |  |  |  |  |
| 16967031 | PAICS | -1.64 |  |  |  |  |  |  |
| 16733104 | CHEK1 | -1.64 |  |  |  |  |  |  |
| 16838359 | BIRC5 | -1.64 |  |  |  |  |  |  |
| 16757687 | RFC5 | -1.64 |  |  |  |  |  |  |
| 17016043 | MBOAT1 | -1.64 |  |  |  |  |  |  |
| 17092688 | HAUS6 | -1.64 |  |  |  |  |  |  |
| 16935517 | CENPM | -1.64 |  |  |  |  |  |  |
| 16699739 | PARP1 | -1.64 |  |  |  |  |  |  |
| 16673983 | DARS2 | -1.64 |  |  |  |  |  |  |
| 16818600 | ORC6 | -1.65 |  |  |  |  |  |  |
| 16669212 | TTF2 | -1.65 |  |  |  |  |  |  |
| 16960271 | HLTF | -1.65 |  |  |  |  |  |  |
| 16987298 | ANKRD32 | -1.65 |  |  |  |  |  |  |
| 17012632 | ENPP1 | -1.65 |  |  |  |  |  |  |
| 16868576 | DNMT1 | -1.66 |  |  |  |  |  |  |
| 17018685 | PPIL1 | -1.66 |  |  |  |  |  |  |
| 17086193 | PSAT1 | -1.66 |  |  |  |  |  |  |
| 16736807 | CCDC34 | -1.66 |  |  |  |  |  |  |
| 16855820 | RTTN | -1.66 |  |  |  |  |  |  |
| 16869624 | DDX39A | -1.66 |  |  |  |  |  |  |
| 16954707 | POC1A | -1.67 |  |  |  |  |  |  |
| 16973263 |  | -1.67 |  |  |  |  |  |  |
| 17016383 | HIST1H4D | -1.67 |  |  |  |  |  |  |
| 16697196 | FAM129A | -1.67 |  |  |  |  |  |  |
| 16809596 | RAB27A | -1.67 |  |  |  |  |  |  |
| 17102230 | POLA1 | -1.68 |  |  |  |  |  |  |
| 16810933 | TIPIN | -1.68 |  |  |  |  |  |  |
| 16779720 | MZT1 | -1.68 |  |  |  |  |  |  |
| 16979060 | ZGRF1 | -1.68 |  |  |  |  |  |  |
| 17084352 | CYP4F26P | -1.69 |  |  |  |  |  |  |
| 16729298 | ACER3 | -1.69 |  |  |  |  |  |  |
| 16866951 | LMNB2 | -1.69 |  |  |  |  |  |  |
| 16762661 | PTHLH | -1.69 |  |  |  |  |  |  |
| 16906440 | OSGEPL1 | -1.69 |  |  |  |  |  |  |
| 16857192 | CHAF1A | -1.69 |  |  |  |  |  |  |
| 16668960 | DCLRE1B | -1.70 |  |  |  |  |  |  |
| 16795304 | CEP128 | -1.70 |  |  |  |  |  |  |
| 16846864 | MMD | -1.71 |  |  |  |  |  |  |
| 16775014 | CKAP2 | -1.71 |  |  |  |  |  |  |
| 16783644 | PNN | -1.71 |  |  |  |  |  |  |
| 17100317 | SAPCD2 | -1.72 |  |  |  |  |  |  |
| 16658987 |  | -1.72 |  |  |  |  |  |  |
| 17049461 | POP7 | -1.72 |  |  |  |  |  |  |
| 17004273 | NQO2 | -1.72 |  |  |  |  |  |  |
| 16829764 | GSG2 | -1.72 |  |  |  |  |  |  |
| 16885411 | GPR17 | -1.72 |  |  |  |  |  |  |
| 17070381 | LRRCC1 | -1.72 |  |  |  |  |  |  |
| 16837729 | NUP85 | -1.72 |  |  |  |  |  |  |
| 16785316 | MTHFD1 | -1.73 |  |  |  |  |  |  |
| 17009862 | PRIM2 | -1.73 |  |  |  |  |  |  |
| 17093595 | FANCG | -1.73 |  |  |  |  |  |  |
| 16803562 | CHRNA5 | -1.73 |  |  |  |  |  |  |
| 16718022 | PCGF6 | -1.74 |  |  |  |  |  |  |
| 16925239 | DONSON | -1.74 |  |  |  |  |  |  |
| 16860418 | CCNE1 | -1.74 |  |  |  |  |  |  |
| 16703452 | PDSS1 | -1.74 |  |  |  |  |  |  |
| 17068385 | GINS4 | -1.74 |  |  |  |  |  |  |
| 16708097 | R3HCC1L | -1.75 |  |  |  |  |  |  |
| 17103413 | SUV39H1 | -1.75 |  |  |  |  |  |  |
| 16773840 | BRCA2 | -1.76 |  |  |  |  |  |  |
| 17094998 | C9orf40 | -1.76 |  |  |  |  |  |  |
| 16705074 | TFAM | -1.76 |  |  |  |  |  |  |
| 16821869 | CDT1 | -1.77 |  |  |  |  |  |  |
| 16833449 | PIGW | -1.77 |  |  |  |  |  |  |
| 17069976 | KCNB2 | -1.77 |  |  |  |  |  |  |
| 16823229 | PKMYT1 | -1.78 |  |  |  |  |  |  |
| 16877956 | CENPA | -1.79 |  |  |  |  |  |  |
| 16821239 | CENPN | -1.79 |  |  |  |  |  |  |
| 16969473 | GSTCD | -1.79 |  |  |  |  |  |  |
| 16845349 | BRCA1 | -1.80 |  |  |  |  |  |  |
| 16766403 | TMEM194A | -1.80 |  |  |  |  |  |  |
| 16769481 | ALDH1L2 | -1.81 |  |  |  |  |  |  |
| 17009809 | KIAA1586 | -1.81 |  |  |  |  |  |  |
| 16767851 | E2F7 | -1.81 |  |  |  |  |  |  |
| 16808876 | CEP152 | -1.81 |  |  |  |  |  |  |
| 17016503 | HIST1H3I | -1.82 |  |  |  |  |  |  |
| 16774082 | EXOSC8 | -1.82 |  |  |  |  |  |  |
| 16819678 | GINS3 | -1.83 |  |  |  |  |  |  |
| 16756960 | FAM216A | -1.83 |  |  |  |  |  |  |
| 17012888 | PEX7 | -1.83 |  |  |  |  |  |  |
| 16755928 | PARPBP | -1.84 |  |  |  |  |  |  |
| 16687618 | DHCR24 | -1.84 |  |  |  |  |  |  |
| 16846218 | HOXB2 | -1.85 |  |  |  |  |  |  |
| 16948021 | ECT2 | -1.85 |  |  |  |  |  |  |
| 16984032 | SKP2 | -1.85 |  |  |  |  |  |  |
| 16919044 | RBL1 | -1.85 |  |  |  |  |  |  |
| 17086784 | CENPP | -1.85 |  |  |  |  |  |  |
| 16914315 | UBE2C | -1.86 |  |  |  |  |  |  |
| 16875763 | UBE2S | -1.86 |  |  |  |  |  |  |
| 16736638 | E2F8 | -1.87 |  |  |  |  |  |  |
| 17112729 | TIMM8A | -1.87 |  |  |  |  |  |  |
| 16832852 | ATAD5 | -1.88 |  |  |  |  |  |  |
| 16842103 | SHMT1 | -1.88 |  |  |  |  |  |  |
| 16667037 | CDC7 | -1.89 |  |  |  |  |  |  |
| 16798810 |  | -1.89 |  |  |  |  |  |  |
| 16929573 | MCM5 | -1.90 |  |  |  |  |  |  |
| 16721535 | ZNF215 | -1.91 |  |  |  |  |  |  |
| 16746379 | NCAPD3 | -1.92 |  |  |  |  |  |  |
| 16692724 | ANP32E | -1.94 |  |  |  |  |  |  |
| 17027710 | TCF19 | -1.95 |  |  |  |  |  |  |
| 16835797 | EME1 | -1.96 |  |  |  |  |  |  |
| 16783047 | G2E3 | -1.97 |  |  |  |  |  |  |
| 16692632 | HIST2H2BE | -1.97 |  |  |  |  |  |  |
| 17109211 | FANCB | -1.97 |  |  |  |  |  |  |
| 16987125 | POLR3G | -1.97 |  |  |  |  |  |  |
| 16985614 | CENPH | -1.98 |  |  |  |  |  |  |
| 17086634 | CKS2 | -1.98 |  |  |  |  |  |  |
| 17079220 | RAD54B | -1.98 |  |  |  |  |  |  |
| 16665447 | USP1 | -1.98 |  |  |  |  |  |  |
| 16792381 | MIS18BP1 | -1.98 |  |  |  |  |  |  |
| 17076103 | TEX15 | -1.99 |  |  |  |  |  |  |
| 16729557 | DDIAS | -1.99 |  |  |  |  |  |  |
| 16880057 | CHAC2 | -1.99 |  |  |  |  |  |  |
| 16701689 |  | -2.00 |  |  |  |  |  |  |
| 16667206 | CCDC18 | -2.00 |  |  |  |  |  |  |
| 16750761 | TROAP | -2.00 |  |  |  |  |  |  |
| 17002912 | BOD1 | -2.01 |  |  |  |  |  |  |
| 16810543 | KIAA0101 | -2.01 |  |  |  |  |  |  |
| 16920548 | AURKA | -2.01 |  |  |  |  |  |  |
| 16725806 | INCENP | -2.02 |  |  |  |  |  |  |
| 16664243 | RAD54L | -2.02 |  |  |  |  |  |  |
| 16671503 | CKS1B | -2.03 |  |  |  |  |  |  |
| 16992096 | SPDL1 | -2.03 |  |  |  |  |  |  |
| 17020019 | MCM3 | -2.05 |  |  |  |  |  |  |
| 16703478 | MASTL | -2.05 |  |  |  |  |  |  |
| 17049714 | LOC101927746 | -2.05 |  |  |  |  |  |  |
| 16817824 | CORO1A | -2.05 |  |  |  |  |  |  |
| 16953279 | CDC25A | -2.06 |  |  |  |  |  |  |
| 16947556 | SMC4 | -2.06 |  |  |  |  |  |  |
| 17093397 | KIF24 | -2.07 |  |  |  |  |  |  |
| 17016372 | HIST1H1C | -2.08 |  |  |  |  |  |  |
| 17058617 | RFC2 | -2.08 |  |  |  |  |  |  |
| 17016390 | HIST1H2BG | -2.08 |  |  |  |  |  |  |
| 16986065 | TMEM171 | -2.08 |  |  |  |  |  |  |
| 16745236 | H2AFX | -2.08 |  |  |  |  |  |  |
| 16721126 | RRM1 | -2.09 |  |  |  |  |  |  |
| 16829369 | FANCA | -2.10 |  |  |  |  |  |  |
| 17068782 | MCM4 | -2.11 |  |  |  |  |  |  |
| 16755692 | GAS2L3 | -2.11 |  |  |  |  |  |  |
| 17005560 | HIST1H4I | -2.12 |  |  |  |  |  |  |
| 16931225 | RIBC2 | -2.12 |  |  |  |  |  |  |
| 16774053 | CCNA1 | -2.12 |  |  |  |  |  |  |
| 16913681 | FAM83D | -2.14 |  |  |  |  |  |  |
| 17060412 | MCM7 | -2.14 |  |  |  |  |  |  |
| 16687188 | ORC1 | -2.14 |  |  |  |  |  |  |
| 16924966 | MIS18A | -2.15 |  |  |  |  |  |  |
| 16962493 | RFC4 | -2.16 |  |  |  |  |  |  |
| 16668079 | GPSM2 | -2.18 |  |  |  |  |  |  |
| 16836492 | PRR11 | -2.18 |  |  |  |  |  |  |
| 16687418 | NDC1 | -2.18 |  |  |  |  |  |  |
| 16670383 | HIST2H2AA4 | -2.19 |  |  |  |  |  |  |
| 16858386 | LDLR | -2.22 |  |  |  |  |  |  |
| 16877473 | GEN1 | -2.22 |  |  |  |  |  |  |
| 17012379 | CENPW | -2.22 |  |  |  |  |  |  |
| 16815090 | CCNF | -2.22 |  |  |  |  |  |  |
| 16858714 | RNASEH2A | -2.24 |  |  |  |  |  |  |
| 16732960 | CCDC15 | -2.25 |  |  |  |  |  |  |
| 16857258 | UHRF1 | -2.25 |  |  |  |  |  |  |
| 16964000 | TACC3 | -2.26 |  |  |  |  |  |  |
| 16669422 |  | -2.28 |  |  |  |  |  |  |
| 16690067 | SASS6 | -2.29 |  |  |  |  |  |  |
| 17005582 | HIST1H2BF | -2.30 |  |  |  |  |  |  |
| 16775324 | BORA | -2.30 |  |  |  |  |  |  |
| 16887810 | ZAK | -2.30 |  |  |  |  |  |  |
| 17064679 | XRCC2 | -2.31 |  |  |  |  |  |  |
| 16773946 | RFC3 | -2.31 |  |  |  |  |  |  |
| 16771067 | CIT | -2.33 |  |  |  |  |  |  |
| 17005396 | GMNN | -2.33 |  |  |  |  |  |  |
| 17057718 | FIGNL1 | -2.34 |  |  |  |  |  |  |
| 16714504 | ZWINT | -2.35 |  |  |  |  |  |  |
| 16957951 | POLQ | -2.36 |  |  |  |  |  |  |
| 16909700 | HJURP | -2.36 |  |  |  |  |  |  |
| 16918445 | E2F1 | -2.36 |  |  |  |  |  |  |
| 17016509 |  | -2.37 |  |  |  |  |  |  |
| 16758336 | KNTC1 | -2.38 |  |  |  |  |  |  |
| 16760048 | FOXM1 | -2.39 |  |  |  |  |  |  |
| 17077826 | MYBL1 | -2.39 |  |  |  |  |  |  |
| 16832429 | TMEM97 | -2.39 |  |  |  |  |  |  |
| 16804902 | BLM | -2.39 |  |  |  |  |  |  |
| 16996722 | CENPK | -2.44 |  |  |  |  |  |  |
| 16736891 | KIF18A | -2.44 |  |  |  |  |  |  |
| 16931384 | GTSE1 | -2.45 |  |  |  |  |  |  |
| 16918976 | DSN1 | -2.46 |  |  |  |  |  |  |
| 17086167 | CEP78 | -2.47 |  |  |  |  |  |  |
| 16804631 | TICRR | -2.48 |  |  |  |  |  |  |
| 17080749 | ATAD2 | -2.49 |  |  |  |  |  |  |
| 17024980 | FBXO5 | -2.50 |  |  |  |  |  |  |
| 17104484 | KIF4A | -2.50 |  |  |  |  |  |  |
| 17020787 | MB21D1 | -2.52 |  |  |  |  |  |  |
| 16845794 | KIF18B | -2.53 |  |  |  |  |  |  |
| 16877019 | RRM2 | -2.53 |  |  |  |  |  |  |
| 16673557 | C1orf112 | -2.54 |  |  |  |  |  |  |
| 16663958 | KIF2C | -2.54 |  |  |  |  |  |  |
| 16784299 | CDKN3 | -2.56 |  |  |  |  |  |  |
| 16760621 | CDCA3 | -2.56 |  |  |  |  |  |  |
| 16714998 | DNA2 | -2.57 |  |  |  |  |  |  |
| 17067332 | ESCO2 | -2.58 |  |  |  |  |  |  |
| 16692580 | HIST2H3PS2 | -2.62 |  |  |  |  |  |  |
| 16799426 | BUB1B | -2.62 |  |  |  |  |  |  |
| 16927052 | CDC45 | -2.62 |  |  |  |  |  |  |
| 16995938 | C5orf34 | -2.64 |  |  |  |  |  |  |
| 16817647 | KIF22 | -2.64 |  |  |  |  |  |  |
| 16793190 | WDHD1 | -2.65 |  |  |  |  |  |  |
| 16945101 | MCM2 | -2.65 |  |  |  |  |  |  |
| 16766318 | PRIM1 | -2.65 |  |  |  |  |  |  |
| 16802204 | ZWILCH | -2.66 |  |  |  |  |  |  |
| 16982024 | CENPU | -2.66 |  |  |  |  |  |  |
| 16937505 | FANCD2 | -2.67 |  |  |  |  |  |  |
| 17016496 | HIST1H2AK | -2.68 |  |  |  |  |  |  |
| 16787430 | CALM1 | -2.70 |  |  |  |  |  |  |
| 16834381 | TUBG1 | -2.71 |  |  |  |  |  |  |
| 16688386 | DEPDC1 | -2.71 |  |  |  |  |  |  |
| 16996545 | DEPDC1B | -2.73 |  |  |  |  |  |  |
| 16685165 | CLSPN | -2.74 |  |  |  |  |  |  |
| 16889251 | SGOL2 | -2.75 |  |  |  |  |  |  |
| 16779546 | DIAPH3 | -2.77 |  |  |  |  |  |  |
| 16979389 | MAD2L1 | -2.77 |  |  |  |  |  |  |
| 16840902 | AURKB | -2.79 |  |  |  |  |  |  |
| 16707695 | HELLS | -2.80 |  |  |  |  |  |  |
| 16985599 | CCNB1 | -2.80 |  |  |  |  |  |  |
| 16847432 | BRIP1 | -2.82 |  |  |  |  |  |  |
| 17096205 | ZNF367 | -2.82 |  |  |  |  |  |  |
| 17038792 | KIFC1 | -2.82 |  |  |  |  |  |  |
| 16698023 | UBE2T | -2.84 |  |  |  |  |  |  |
| 16913957 | MYBL2 | -2.84 |  |  |  |  |  |  |
| 16830173 | FAM64A | -2.88 |  |  |  |  |  |  |
| 17087716 | SMC2 | -2.89 |  |  |  |  |  |  |
| 16663514 | CDC20 | -2.89 |  |  |  |  |  |  |
| 16807605 | OIP5 | -2.91 |  |  |  |  |  |  |
| 16849379 | TK1 | -2.93 |  |  |  |  |  |  |
| 17064939 | NCAPG2 | -2.93 |  |  |  |  |  |  |
| 16705159 | CDK1 | -2.98 |  |  |  |  |  |  |
| 17105401 | CENPI | -2.99 |  |  |  |  |  |  |
| 16972616 | NEIL3 | -3.00 |  |  |  |  |  |  |
| 16982635 | TRIP13 | -3.00 |  |  |  |  |  |  |
| 16815905 | RMI2 | -3.00 |  |  |  |  |  |  |
| 16903090 | MCM6 | -3.02 |  |  |  |  |  |  |
| 16692636 | HIST2H2AB | -3.04 |  |  |  |  |  |  |
| 16686796 | STIL | -3.07 |  |  |  |  |  |  |
| 16991859 | HMMR | -3.08 |  |  |  |  |  |  |
| 16751709 | ESPL1 | -3.11 |  |  |  |  |  |  |
| 16697695 | KIF14 | -3.14 |  |  |  |  |  |  |
| 16911212 | MCM8 | -3.14 |  |  |  |  |  |  |
| 16679411 | EXO1 | -3.16 |  |  |  |  |  |  |
| 16813342 | PRC1 | -3.17 |  |  |  |  |  |  |
| 16725735 | FEN1 | -3.18 |  |  |  |  |  |  |
| 17080595 | DSCC1 | -3.21 |  |  |  |  |  |  |
| 17000439 | CDC25C | -3.21 |  |  |  |  |  |  |
| 17047965 | DBF4 | -3.25 |  |  |  |  |  |  |
| 16799637 | RAD51 | -3.27 |  |  |  |  |  |  |
| 16694617 | IQGAP3 | -3.27 |  |  |  |  |  |  |
| 17016363 | HIST1H3B | -3.29 |  |  |  |  |  |  |
| 16826160 | SHCBP1 | -3.30 |  |  |  |  |  |  |
| 16988703 | LMNB1 | -3.33 |  |  |  |  |  |  |
| 16938296 | SGOL1-AS1 | -3.38 |  |  |  |  |  |  |
| 16804559 | FANCI | -3.41 |  |  |  |  |  |  |
| 16842673 | SPAG5 | -3.46 |  |  |  |  |  |  |
| 16702685 | SUV39H2 | -3.46 |  |  |  |  |  |  |
| 16817017 | PLK1 | -3.46 |  |  |  |  |  |  |
| 17067102 | CDCA2 | -3.46 |  |  |  |  |  |  |
| 16800355 | WDR76 | -3.47 |  |  |  |  |  |  |
| 16707221 | KIF20B | -3.47 |  |  |  |  |  |  |
| 16662648 | CDCA8 | -3.50 |  |  |  |  |  |  |
| 16698984 | NEK2 | -3.52 |  |  |  |  |  |  |
| 16868838 | SPC24 | -3.59 |  |  |  |  |  |  |
| 16798801 | ARHGAP11B | -3.60 |  |  |  |  |  |  |
| 16677425 | CENPF | -3.65 |  |  |  |  |  |  |
| 16912379 | TPX2 | -3.68 |  |  |  |  |  |  |
| 16792519 | POLE2 | -3.70 |  |  |  |  |  |  |
| 16979515 | CCNA2 | -3.74 |  |  |  |  |  |  |
| 17016366 | HIST1H2AB | -3.75 |  |  |  |  |  |  |
| 16673154 | NUF2 | -3.75 |  |  |  |  |  |  |
| 17005589 | HIST1H2AE | -3.76 |  |  |  |  |  |  |
| 16882975 | NCAPH | -3.77 |  |  |  |  |  |  |
| 16702571 | MCM10 | -3.80 |  |  |  |  |  |  |
| 16957170 | KIAA1524 | -3.81 |  |  |  |  |  |  |
| 16777278 | SKA3 | -3.85 |  |  |  |  |  |  |
| 16747014 | RAD51AP1 | -3.86 |  |  |  |  |  |  |
| 16793225 | DLGAP5 | -3.88 |  |  |  |  |  |  |
| 16677201 | DTL | -3.91 |  |  |  |  |  |  |
| 16719515 | MKI67 | -3.95 |  |  |  |  |  |  |
| 17010552 | TTK | -3.95 |  |  |  |  |  |  |
| 16951485 | SGOL1 | -3.98 |  |  |  |  |  |  |
| 16834056 | CDC6 | -3.98 |  |  |  |  |  |  |
| 16991460 | KIF4B | -3.99 |  |  |  |  |  |  |
| 16869588 | ASF1B | -4.02 |  |  |  |  |  |  |
| 16844312 | TOP2A | -4.09 |  |  |  |  |  |  |
| 16852312 | SKA1 | -4.18 |  |  |  |  |  |  |
| 17079293 | CCNE2 | -4.21 |  |  |  |  |  |  |
| 16799598 | CASC5 | -4.22 |  |  |  |  |  |  |
| 16978568 | CENPE | -4.33 |  |  |  |  |  |  |
| 16809748 | MNS1 | -4.35 |  |  |  |  |  |  |
| 16707551 | CEP55 | -4.43 |  |  |  |  |  |  |
| 16799793 | NUSAP1 | -4.45 |  |  |  |  |  |  |
| 16904780 | SPC25 | -4.47 |  |  |  |  |  |  |
| 16971573 | MND1 | -4.48 |  |  |  |  |  |  |
| 16697544 | ASPM | -4.50 |  |  |  |  |  |  |
| 17084904 | MELK | -4.54 |  |  |  |  |  |  |
| 16850517 | NDC80 | -4.56 |  |  |  |  |  |  |
| 16912192 | GINS1 | -4.59 |  |  |  |  |  |  |
| 16798919 | ARHGAP11A | -4.69 |  |  |  |  |  |  |
| 16970563 | PLK4 | -4.70 |  |  |  |  |  |  |
| 16901755 | BUB1 | -4.71 |  |  |  |  |  |  |
| 16828886 | GINS2 | -4.73 |  |  |  |  |  |  |
| 17045198 | ANLN | -4.75 |  |  |  |  |  |  |
| 16901957 | CKAP2L | -4.75 |  |  |  |  |  |  |
| 16801557 | CCNB2 | -4.87 |  |  |  |  |  |  |
| 16939960 | KIF15 | -4.89 |  |  |  |  |  |  |
| 16707468 | KIF11 | -4.89 |  |  |  |  |  |  |
| 16965346 | NCAPG | -4.99 |  |  |  |  |  |  |
| 17005858 | HIST1H2AI | -5.00 |  |  |  |  |  |  |
| 16989636 | KIF20A | -5.21 |  |  |  |  |  |  |
| 16670387 | HIST2H3A | -5.27 |  |  |  |  |  |  |
| 17016369 | HIST1H2BB | -5.64 |  |  |  |  |  |  |
| 16802519 | KIF23 | -5.68 |  |  |  |  |  |  |
| 17075776 | PBK | -5.82 |  |  |  |  |  |  |
| 17005865 | HIST1H2BM | -6.49 |  |  |  |  |  |  |
| 17016486 | HIST1H2BL | -6.63 |  |  |  |  |  |  |
| 16725041 | FAM111B | -7.41 |  |  |  |  |  |  |
| 17016499 | HIST1H1B | -7.66 |  |  |  |  |  |  |

*TICAE cells were analyzed at the indicated time points after irradiation with a single X-ray dose of 2 Gy. Fold changes are shown compared to sham irradiation, as described in Materials and Methods (n = 3).
