# Supplementary material for: Functional Gene Analysis Reveals Cell Cycle Changes and Inflammation in Endothelial Cells Irradiated with a Single X-ray Dose
Source: Front Pharmacol. 2017 Apr 25;8:213. doi: 10.3389/fphar.2017.00213 (PMC5404649; doi:10.3389/fphar.2017.00213)
Supplement: Supplementary file 9 [file Table2.DOCX]

Supplementary Material

**Functional Gene Analysis Reveals Cell Cycle Changes and Inflammation in Endothelial Cells Irradiated with a Single X-ray Dose**

**Bjorn Baselet^1,2^, Niels Belmans^1,3^, Emma Coninx^1^, Donna Lowe^4^, Ann Janssen^1^, Arlette Michaux^1^, Kevin Tabury^1,5^, Kenneth Raj^4^, Roel Quintens^1^, Abderrafi Mohammed Benotmane^1,$^, Sarah Baatout^1,6,$^, Pierre Sonveaux^2,$^ An Aerts^1,^** $**^,*^**

*** Correspondence:** An Aerts: [an.aerts@sckcen.be](mailto:an.aerts@sckcen.be)

**Supplementary table 2. Differentially expressed genes TICAE cells irradiated with a single X-ray dose of 0.5 Gy.***

| Day 1 | | | Day 7 | | | Day 14 | | |
| --- | --- | --- | --- | --- | --- | --- | --- | --- |
| 0.5 Gy vs. 0 Gy | | | 0.5 Gy vs. 0 Gy | | | 0.5 Gy vs. 0 Gy | | |
| Probeset ID | Gene symbol | Fold change | Probeset ID | Gene symbol | Fold change | Probeset ID | Gene symbol | Fold change |
| 17042857 | AC091729.8 | 2.17 | 16760257 | VWF | -1.60044 | 16997676 | MTRNR2L2 | 1.8933 |
| 16974115 |  | 2.15 | 16787362 | RN7SKP255 | 2.27477 | 16862604 | CD79A | 1.78593 |
| 16988913 | RNU6ATAC10P | 1.98 |  |  |  | 16782050 | MGC40069 | 2.11157 |
| 17073066 | RNA5SP278 | 1.95 |  |  |  | 16787362 | RN7SKP255 | 2.23748 |
| 16744154 | RP11-25I9.2 | 1.95 |  |  |  |  |  |  |
| 17074080 |  | 1.93 |  |  |  |  |  |  |
| 17072723 | RNU1-106P | 1.82 |  |  |  |  |  |  |
| 16905115 |  | 1.79 |  |  |  |  |  |  |
| 17005167 | RNU6-190P | 1.76 |  |  |  |  |  |  |
| 16681370 | ENO1 | 1.76 |  |  |  |  |  |  |
| 16831787 |  | 1.75 |  |  |  |  |  |  |
| 16897446 | RNU6-439P | 1.71 |  |  |  |  |  |  |
| 16660309 | RP3-340N1.2 | 1.66 |  |  |  |  |  |  |
| 16956613 |  | 1.63 |  |  |  |  |  |  |
| 16892863 | AC112715.2 | 1.63 |  |  |  |  |  |  |
| 16997676 | MTRNR2L2 | 1.62 |  |  |  |  |  |  |
| 16753853 | MDM2 | 1.58 |  |  |  |  |  |  |
| 16966685 | SPATA18 | 1.58 |  |  |  |  |  |  |
| 17056072 | SKAP2 | 1.55 |  |  |  |  |  |  |
| 16828833 |  | 1.55 |  |  |  |  |  |  |
| 16991839 | CCNG1 | 1.54 |  |  |  |  |  |  |
| 16696425 | TNFSF4 | 1.52 |  |  |  |  |  |  |
| 16991164 | MYOZ3 | -1.50 |  |  |  |  |  |  |
| 16829153 | SNAI3 | -1.50 |  |  |  |  |  |  |
| 16815090 | CCNF | -1.50 |  |  |  |  |  |  |
| 16692724 | ANP32E | -1.50 |  |  |  |  |  |  |
| 16687418 | NDC1 | -1.50 |  |  |  |  |  |  |
| 16927052 | CDC45 | -1.51 |  |  |  |  |  |  |
| 16669389 | PHGDH | -1.51 |  |  |  |  |  |  |
| 17009794 | BEND6 | -1.53 |  |  |  |  |  |  |
| 16847432 | BRIP1 | -1.53 |  |  |  |  |  |  |
| 16821869 | CDT1 | -1.53 |  |  |  |  |  |  |
| 16911212 | MCM8 | -1.54 |  |  |  |  |  |  |
| 16767851 | E2F7 | -1.54 |  |  |  |  |  |  |
| 16850477 | TYMS | -1.55 |  |  |  |  |  |  |
| 16910501 | DTYMK | -1.55 |  |  |  |  |  |  |
| 16992096 | SPDL1 | -1.55 |  |  |  |  |  |  |
| 16663958 | KIF2C | -1.56 |  |  |  |  |  |  |
| 16838359 | BIRC5 | -1.56 |  |  |  |  |  |  |
| 16909700 | HJURP | -1.57 |  |  |  |  |  |  |
| 16667206 | CCDC18 | -1.57 |  |  |  |  |  |  |
| 16698023 | UBE2T | -1.58 |  |  |  |  |  |  |
| 16779546 | DIAPH3 | -1.58 |  |  |  |  |  |  |
| 17067332 | ESCO2 | -1.58 |  |  |  |  |  |  |
| 16775324 | BORA | -1.58 |  |  |  |  |  |  |
| 16745236 | H2AFX | -1.58 |  |  |  |  |  |  |
| 16747287 | NCAPD2 | -1.59 |  |  |  |  |  |  |
| 16810543 | KIAA0101 | -1.59 |  |  |  |  |  |  |
| 17068782 | MCM4 | -1.59 |  |  |  |  |  |  |
| 17096205 | ZNF367 | -1.60 |  |  |  |  |  |  |
| 16707468 | KIF11 | -1.60 |  |  |  |  |  |  |
| 16857258 | UHRF1 | -1.60 |  |  |  |  |  |  |
| 16858714 | RNASEH2A | -1.60 |  |  |  |  |  |  |
| 16799426 | BUB1B | -1.61 |  |  |  |  |  |  |
| 16804559 | FANCI | -1.61 |  |  |  |  |  |  |
| 16913681 | FAM83D | -1.61 |  |  |  |  |  |  |
| 16667037 | CDC7 | -1.62 |  |  |  |  |  |  |
| 16858386 | LDLR | -1.63 |  |  |  |  |  |  |
| 17068385 | GINS4 | -1.63 |  |  |  |  |  |  |
| 16679411 | EXO1 | -1.64 |  |  |  |  |  |  |
| 16972616 | NEIL3 | -1.64 |  |  |  |  |  |  |
| 17005865 | HIST1H2BM | -1.65 |  |  |  |  |  |  |
| 16750761 | TROAP | -1.65 |  |  |  |  |  |  |
| 16802204 | ZWILCH | -1.66 |  |  |  |  |  |  |
| 17038792 | KIFC1 | -1.66 |  |  |  |  |  |  |
| 17012632 | ENPP1 | -1.66 |  |  |  |  |  |  |
| 16948021 | ECT2 | -1.66 |  |  |  |  |  |  |
| 16985614 | CENPH | -1.67 |  |  |  |  |  |  |
| 17064285 | TMEM176B | -1.68 |  |  |  |  |  |  |
| 16705159 | CDK1 | -1.68 |  |  |  |  |  |  |
| 16688386 | DEPDC1 | -1.68 |  |  |  |  |  |  |
| 16951485 | SGOL1 | -1.69 |  |  |  |  |  |  |
| 16725041 | FAM111B | -1.69 |  |  |  |  |  |  |
| 16736891 | KIF18A | -1.69 |  |  |  |  |  |  |
| 17016366 | HIST1H2AB | -1.70 |  |  |  |  |  |  |
| 16869588 | ASF1B | -1.70 |  |  |  |  |  |  |
| 16739479 | LRRN4CL | -1.71 |  |  |  |  |  |  |
| 16964000 | TACC3 | -1.71 |  |  |  |  |  |  |
| 17105401 | CENPI | -1.71 |  |  |  |  |  |  |
| 16995938 | C5orf34 | -1.71 |  |  |  |  |  |  |
| 17086167 | CEP78 | -1.72 |  |  |  |  |  |  |
| 16834056 | CDC6 | -1.74 |  |  |  |  |  |  |
| 16845794 | KIF18B | -1.74 |  |  |  |  |  |  |
| 17064939 | NCAPG2 | -1.74 |  |  |  |  |  |  |
| 16957951 | POLQ | -1.76 |  |  |  |  |  |  |
| 16662648 | CDCA8 | -1.76 |  |  |  |  |  |  |
| 16784299 | CDKN3 | -1.76 |  |  |  |  |  |  |
| 16760621 | CDCA3 | -1.77 |  |  |  |  |  |  |
| 16817647 | KIF22 | -1.77 |  |  |  |  |  |  |
| 16673154 | NUF2 | -1.78 |  |  |  |  |  |  |
| 17067102 | CDCA2 | -1.78 |  |  |  |  |  |  |
| 16982635 | TRIP13 | -1.78 |  |  |  |  |  |  |
| 16828886 | GINS2 | -1.79 |  |  |  |  |  |  |
| 16912192 | GINS1 | -1.79 |  |  |  |  |  |  |
| 16798919 | ARHGAP11A | -1.80 |  |  |  |  |  |  |
| 16988703 | LMNB1 | -1.80 |  |  |  |  |  |  |
| 16686796 | STIL | -1.80 |  |  |  |  |  |  |
| 16889251 | SGOL2 | -1.81 |  |  |  |  |  |  |
| 16813342 | PRC1 | -1.81 |  |  |  |  |  |  |
| 16840902 | AURKB | -1.82 |  |  |  |  |  |  |
| 17084904 | MELK | -1.83 |  |  |  |  |  |  |
| 16694617 | IQGAP3 | -1.84 |  |  |  |  |  |  |
| 16807605 | OIP5 | -1.84 |  |  |  |  |  |  |
| 16836492 | PRR11 | -1.85 |  |  |  |  |  |  |
| 16931225 | RIBC2 | -1.85 |  |  |  |  |  |  |
| 16690067 | SASS6 | -1.85 |  |  |  |  |  |  |
| 16771067 | CIT | -1.86 |  |  |  |  |  |  |
| 16826160 | SHCBP1 | -1.86 |  |  |  |  |  |  |
| 16760048 | FOXM1 | -1.87 |  |  |  |  |  |  |
| 17087716 | SMC2 | -1.88 |  |  |  |  |  |  |
| 16677201 | DTL | -1.90 |  |  |  |  |  |  |
| 17104484 | KIF4A | -1.90 |  |  |  |  |  |  |
| 16985599 | CCNB1 | -1.90 |  |  |  |  |  |  |
| 16931384 | GTSE1 | -1.90 |  |  |  |  |  |  |
| 16957170 | KIAA1524 | -1.90 |  |  |  |  |  |  |
| 16937505 | FANCD2 | -1.92 |  |  |  |  |  |  |
| 16912379 | TPX2 | -1.93 |  |  |  |  |  |  |
| 16979515 | CCNA2 | -1.93 |  |  |  |  |  |  |
| 16979389 | MAD2L1 | -1.94 |  |  |  |  |  |  |
| 17012379 | CENPW | -2.00 |  |  |  |  |  |  |
| 16913957 | MYBL2 | -2.01 |  |  |  |  |  |  |
| 16978568 | CENPE | -2.03 |  |  |  |  |  |  |
| 16751709 | ESPL1 | -2.05 |  |  |  |  |  |  |
| 16965346 | NCAPG | -2.05 |  |  |  |  |  |  |
| 16904780 | SPC25 | -2.07 |  |  |  |  |  |  |
| 17010552 | TTK | -2.09 |  |  |  |  |  |  |
| 16777278 | SKA3 | -2.09 |  |  |  |  |  |  |
| 16668079 | GPSM2 | -2.09 |  |  |  |  |  |  |
| 16830173 | FAM64A | -2.11 |  |  |  |  |  |  |
| 16707221 | KIF20B | -2.11 |  |  |  |  |  |  |
| 16849379 | TK1 | -2.11 |  |  |  |  |  |  |
| 16719515 | MKI67 | -2.12 |  |  |  |  |  |  |
| 16842673 | SPAG5 | -2.13 |  |  |  |  |  |  |
| 16663514 | CDC20 | -2.14 |  |  |  |  |  |  |
| 16991859 | HMMR | -2.18 |  |  |  |  |  |  |
| 16882975 | NCAPH | -2.18 |  |  |  |  |  |  |
| 16799598 | CASC5 | -2.21 |  |  |  |  |  |  |
| 16799793 | NUSAP1 | -2.21 |  |  |  |  |  |  |
| 16868838 | SPC24 | -2.22 |  |  |  |  |  |  |
| 16850517 | NDC80 | -2.23 |  |  |  |  |  |  |
| 16697695 | KIF14 | -2.26 |  |  |  |  |  |  |
| 16901957 | CKAP2L | -2.26 |  |  |  |  |  |  |
| 16801557 | CCNB2 | -2.26 |  |  |  |  |  |  |
| 16809748 | MNS1 | -2.27 |  |  |  |  |  |  |
| 16939960 | KIF15 | -2.28 |  |  |  |  |  |  |
| 16802519 | KIF23 | -2.29 |  |  |  |  |  |  |
| 16793225 | DLGAP5 | -2.29 |  |  |  |  |  |  |
| 17000439 | CDC25C | -2.30 |  |  |  |  |  |  |
| 17075776 | PBK | -2.32 |  |  |  |  |  |  |
| 16698984 | NEK2 | -2.34 |  |  |  |  |  |  |
| 17049700 | MIR4653 | -2.34 |  |  |  |  |  |  |
| 16817017 | PLK1 | -2.37 |  |  |  |  |  |  |
| 17045198 | ANLN | -2.42 |  |  |  |  |  |  |
| 16875763 | UBE2S | -2.42 |  |  |  |  |  |  |
| 16677425 | CENPF | -2.43 |  |  |  |  |  |  |
| 16697544 | ASPM | -2.49 |  |  |  |  |  |  |
| 16844312 | TOP2A | -2.50 |  |  |  |  |  |  |
| 16707551 | CEP55 | -2.54 |  |  |  |  |  |  |
| 16901755 | BUB1 | -2.69 |  |  |  |  |  |  |
| 16971573 | MND1 | -2.83 |  |  |  |  |  |  |
| 16989636 | KIF20A | -3.14 |  |  |  |  |  |  |
| 16991460 | KIF4B | -3.38 |  |  |  |  |  |  |

*TICAE cells were analyzed at the indicated time points after irradiation with a single X-ray dose of 0.5 Gy. Fold changes are shown compared to sham irradiation, as described in Materials and Methods (n = 3).
