# Supplementary material for: Functional Gene Analysis Reveals Cell Cycle Changes and Inflammation in Endothelial Cells Irradiated with a Single X-ray Dose
Source: Front Pharmacol. 2017 Apr 25;8:213. doi: 10.3389/fphar.2017.00213 (PMC5404649; doi:10.3389/fphar.2017.00213)
Supplement: Supplementary file 10 [file Table3.DOCX]

Supplementary Material

**Functional Gene Analysis Reveals Cell Cycle Changes and Inflammation in Endothelial Cells Irradiated with a Single X-ray Dose**

**Bjorn Baselet^1,2^, Niels Belmans^1,3^, Emma Coninx^1^, Donna Lowe^4^, Ann Janssen^1^, Arlette Michaux^1^, Kevin Tabury^1,5^, Kenneth Raj^4^, Roel Quintens^1^, Abderrafi Mohammed Benotmane^1,$^, Sarah Baatout^1,6,$^, Pierre Sonveaux^2,$^ An Aerts^1,^** $**^,*^**

*** Correspondence:** An Aerts: [an.aerts@sckcen.be](mailto:an.aerts@sckcen.be)

**Supplementary table 3. Differentially expressed genes in TICAE cells irradiated with a single X-ray dose of 0.05 and 0.1 Gy.***

| Day 14 | | | Day 7 | | |
| --- | --- | --- | --- | --- | --- |
| 0.1 Gy vs. 0 Gy | | | 0.05 Gy vs. 0 Gy | | |
| Probeset ID | Gene symbol | Fold change | Probeset ID | Gene symbol | Fold change |
| 16997676 | MTRNR2L2 | 2.02 | 16862604 | CD79A | 1.84 |

*TICAE cells were analyzed at the indicated time points after irradiation with a single X-ray dose of 0.1 and 0.05 Gy. Fold changes are shown compared to sham irradiation, as described in Materials and Methods (n = 3).
